# Reversible Enzymatic Switching of the Oxidation State of a Eu^III/II^ Complex Controls Relaxivity

**DOI:** 10.1021/jacs.5c13447

**Published:** 2025-10-16

**Authors:** Euan T. Sarson, Saul M. Cooper, Adam C. Sedgwick, Amy-Grace Berger, Sophie A. Twigger, Ester M. Hammond, Kylie A. Vincent, Stephen Faulkner

**Affiliations:** † Department of Chemistry, Chemistry Research Laboratory, 6396 University of Oxford, Mansfield Road, Oxford OX1 3TA, United Kingdom; ‡ Department of Oncology, Old Road Campus Research Building, University of Oxford, Oxford OX3 7DQ, United Kingdom

## Abstract

We show that europium trisbipyridyl cryptate exhibits an electrochemically reversible Eu^III/II^ redox couple at −360 mV vs the standard hydrogen electrode (SHE) and is susceptible to reduction or oxidation by a range of redox enzymes or biological small molecule redox agents. Modulation of the europium oxidation state can be used to achieve dramatic changes in the *T*_1_ relaxation time of bulk water in solutions containing these complexes: in the reduced form, the complex exhibits high relaxivity (3.08 mM^–1^ s^–1^ at 499.9 MHz) comparable with that of commercial MRI contrast media, while the oxidized form has negligible relaxivity, illustrating the scope for application in imaging reducing environments in biological systems.

## Graphical abstract

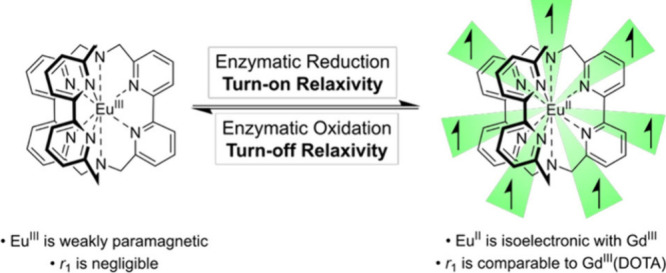

In healthy mammalian cells, redox conditions are tightly regulated by a series of oxidoreductase enzymes and small molecule antioxidants.^1^ Changes in O_2_ level have profound effects on cellular redox balance, with a cascade of biochemical consequences.^2,3^ This is of particular importance in cancer tissue, where poor O_2_ access leads to hypoxia and a reducing environment in cells.^4–7^ Understanding the redox conditions in cancer tissue has medical relevance, as hypoxic tumors are aggressive and therapy-resistant.^8^ Probes for molecular imaging that are activated by reduction offer scope for the selective imaging of hypoxic tissue. Imaging of redox status has been limited largely to fluorophores.^4,9–12^ While fluorescence imaging is relatively inexpensive, it suffers from limited penetration depth (<1 cm)^13^ and the fluorescence emission can be difficult to distinguish from autofluorescence.^14^ Here, we explore the possibility of magnetic resonance imaging (MRI) with selectivity for reducing environments.

MRI offers the potential for whole-body imaging with millimeter resolution through using gradient fields to provide spatial resolution of the NMR signals arising from water protons. The use of paramagnetic metal complexes to enhance water relaxation rates has proved highly effective in clinical imaging.^15^ Gadolinium complexes have dominated this field;^16^ the f^7^ configuration and ^8^S_7/2_ ground state of Gd^III^ gives rapid relaxation of protons on bound water molecules, which are in fast exchange with bulk water. However, Gd^III^ is not susceptible to redox change in aqueous solution.^17^ Eu^II^ is isoelectronic with Gd^III^ and has been explored as an alternative contrast medium.^18–20^ The Eu _aq_^3+/2+^ couple is at −0.35 V to −0.43 V, depending on conditions,^21,22^ suggesting that Eu^II^ should be accessible in the biochemical conditions associated with hypoxia given that the redox potential of hypoxic human adenocarcinoma cells (A549) has been measured as approximately −0.33 to −0.44 V vs NHE.^23^ Eu^III^ is an f^6^ ion with a ^7^F_0_ ground state and limited thermal population of its ^7^F_1_ and ^7^F_2_ excited states. As such, Eu^III^ complexes have very low magnetic moments. Hence, manipulation of the Eu^III/II^ redox couple offers opportunities for redox-responsive imaging. However, the ligands used in clinical contrast agents shift the Eu^III/II^ couple considerably negative relative to the aquo ions, meaning that simply replacing gadolinium with europium will not achieve the goal of stabilizing Eu^II^ under hypoxic conditions.

Allen and co-workers administered Eu^II^ complexes in mice and showed that it was possible to exploit slow oxidation in physoxic tissue (i.e., tissue in which *p*_O_2__ ≈ 3%–7.4%)^24^ to achieve image contrast with hypoxic tissue where the Eu^II^ species is more stable.^25^ The alternative approach of generating Eu^II^ species in hypoxic tissue from Eu^III^ complexes has not been explored but offers the advantage of only highlighting hypoxic regions, as well as the ease of administering air-stable Eu^III^ complexes. Here, we explore tuning the ligand environment in order to define systems with a Eu^III/II^ potential compatible with reduction of the lanthanide in hypoxic tissue. We synthesized a variety of europium complexes and examined their electrochemical behaviors by cyclic voltammetry to identify those with suitable *E*_1/2_ at physiological pH. The NAD­(P)^+^/NAD­(P)H couple (−0.32 V)^26^ is an important cellular redox couple, and we identify a complex susceptible to NADH-dependent enzyme-mediated reduction, which dramatically enhances the *T*_1_ relaxivity, as a consequence of the formation of Eu^II^.

Schemes S1–S9 in the Supporting Information (SI) outline the syntheses of **Eu** ^**III**^ **(DOTA)**, **Eu** ^**III**^ **(TAC)**, **Eu** ^**III**^ **(DTDCC)**, **Eu** ^**III**^ **(TPC)**, **Eu** ^**III/II**^ **(LBC)**, **Eu** ^**II**^ **(221)**, **Eu** ^**II**^ **(222)**, and **Eu** ^**II**^ **(222B)**, respectively. **Eu­(TAC)** and **Eu­(DTDCC)** are novel complexes and the other complexes were synthesized according to adapted literature procedures.^27–29^ Cyclic voltammetry (CV) was performed on all complexes at a glassy carbon working electrode at pH 7.4 (Figures S2–S9). The midpoint potentials for the Eu^III/II^ couple in the complexes are summarized in Figure 1. **Eu­(DOTA)**, **Eu­(TAC)**, and **Eu­(DTDCC)** show potentials significantly negative of the aquo ion couple, indicating that such complexes would not be reduced, even in hypoxic tissue. As noted previously, **Eu­(221)**, **Eu­(222)**, and **Eu­(222B)** show potentials shifted significantly positive of the aquo ion couple (*E*_1/2_ = −0.191, +0.032, and +0.139 V, respectively) indicative of greater stabilization of Eu^II^, relative to Eu^III^, such that they would favor the reduced form even under physoxic conditions, but the complex electrochemistry is suggestive of the presence of free Eu_aq_^3+/2+^.^30–32^ Notably, **Eu­(LBC)** and **Eu­(TPC)** show reversible Eu^III/II^ couples (see Figures S5 and S6), at *E*_1/2_ = −0.360 V and −0.389 V, respectively, suggesting that they would be reduced in hypoxic, but not physoxic, environments. The green bar on Figure 1 indicates the range of potentials observed in hypoxic human A549 cells.^23^ While **Eu­(TPC)** is kinetically labile, the kinetic stability of **Eu** ^**III**^ **(LBC)** has been established across a range of biological assays.^33,34^ At a scan rate of 1 mV/s, **Eu** ^**III**^ **(LBC)** shows a peak separation of 68 mV in the cyclic voltammogram, close to the expected 59 mV separation for a reversible, diffusion-controlled one-electron process. Figure S10 shows how the voltammograms vary with scan rate, including the expected linear dependence of peak current on the square root of the scan rate (Figure S11). We therefore focused on **Eu­(LBC)** for further study. The Lehn Bipyridyl Cryptand (**LBC**) is well-established as a luminescent lanthanide complex and is widely employed in bioassay.^27,33,34^ The luminescence lifetimes (0.34 ms in H_2_O and 1.70 ms in D_2_O)^27^ and inner sphere hydration (*q* = 2.5)^27^ of **Eu** ^**III**^ **(LBC)** also indicate that LBC complexes may be useful in MRI, since the presence of exchangeable inner sphere water will enhance the relaxometric properties of the complex. In the case of this system, a degree of caution is necessary, since the inner sphere solvation of **Eu** ^**II**^ **(LBC)** may differ from that of the **Eu** ^**III**^ **(LBC)** system. However, a larger cation would be expected to be unlikely to display reduced solvation.

**Figure 1:**
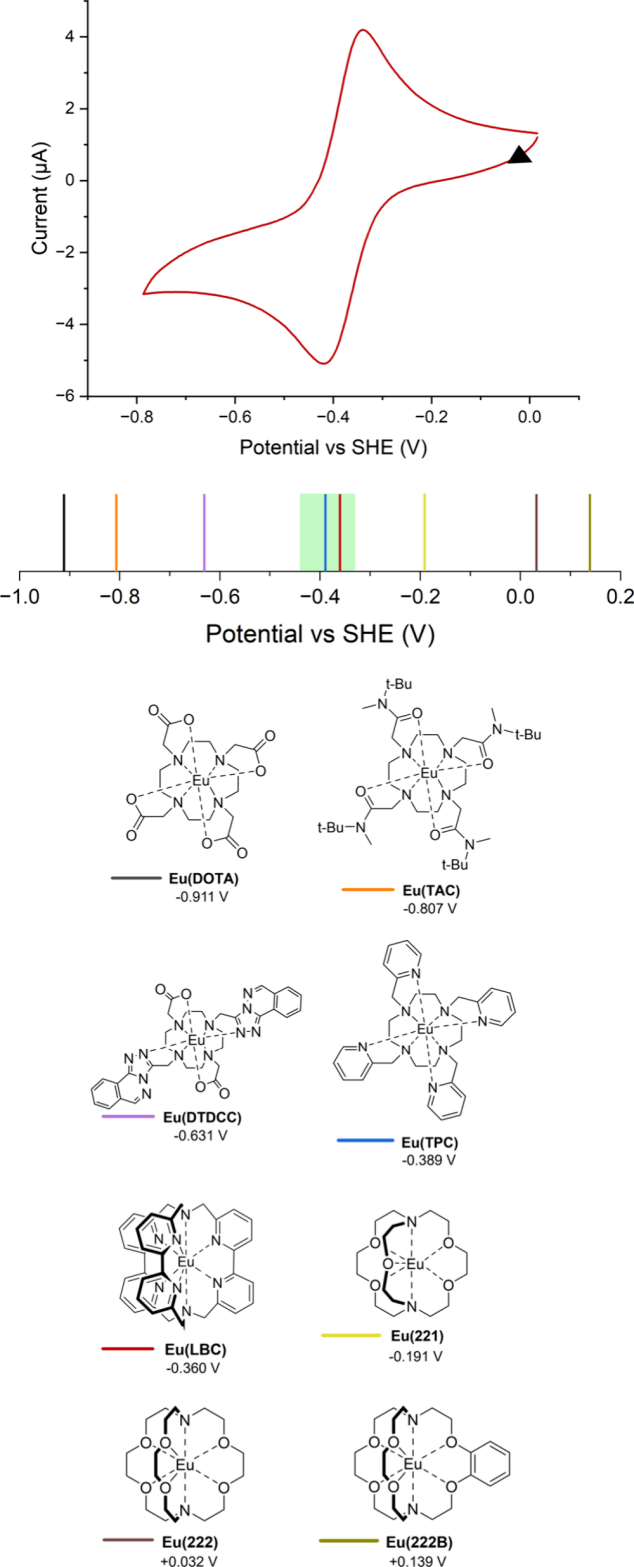
Cyclic voltammogram of **Eu­(LBC)** at a glassy carbon electrode (1 mM in Tris (50 mM with 50 mM NaCl)) at pH 7.4, scan rate = 0.02 V/s. Midpoint potentials (*E*_1/2_) of the Eu^III/II^ couple of various europium complexes measured in H_2_O at pH 7.4 (50 mM Tris, 50 mM NaCl). The region highlighted in green shows the potential range previously reported for hypoxic A549 cells.^23^

We studied the relaxometric properties of **Eu­(LBC)** in aqueous solution at pH 7.4, and determined the longitudinal relaxation time, *T*_1_, for water protons in solutions containing the complexes. Figure 2 shows a plot of 1/*T*_1_ vs concentration of the complex, where the slope indicates ^1^H relaxivity, *r*_1_. The relaxivity for **Eu** ^**II**^ **(LBC)** was found to be 3.08 ± 0.03 mM­(Eu)^−1^ s^–1^ at 11.75 T, which is comparable to that of the commercial MRI contrast agent **Gd** ^**III**^ **(DOTA)** (Dotarem) under the same conditions (Table S5). By contrast, **Eu** ^**III**^ **(LBC)** was found to have negligible relaxivity, 0.001 ± 0.004 mM­(Eu)^−1^ s^–1^. This indicates that redox-induced amplification of relaxivity could potentially be achieved with the **Eu­(LBC)** system. The observed relaxivity of **Eu** ^**II**^ **(LBC)** confirms the supposition that the complex is associated with inner sphere water in rapid exchange.

**Figure 2:**
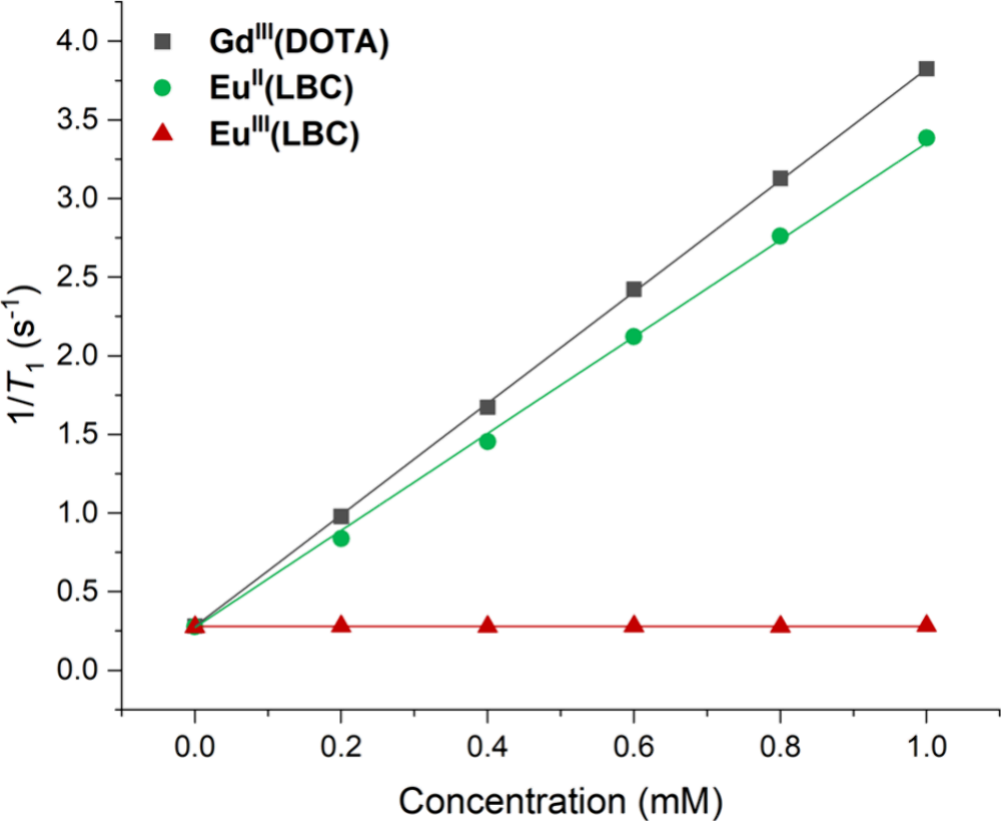
1/*T*_1_ versus concentration plot for **Eu** ^**III**^ **(LBC)**, **Eu** ^**II**^ **(LBC)**, and **Gd** ^**III**^ **(DOTA)** from which the *r*_1_ values extracted from the gradients of the linear fits; *T*_1_ values were measured in degassed H_2_O at pH 7.4 (50 mM Tris, 50 mM NaCl) using an inversion recovery sequence on an 11.75 T (499.9 MHz) NMR spectrometer.

We then explored the scope for reduction of **Eu** ^**III**^ **(LBC)** by biomolecules, using *T*_1_ of water protons as an indicator for reduction of the europium complex (see Tables S6–S13 and S22). With reduced cofactors that are upregulated in hypoxia, NAD­(P)H and GSH,^6,7^ no significant change in *T*_1_ was observed (Tables S6, S9, and S22). This is consistent with the fact that these cofactors typically act as hydride transfer agents, requiring enzymes to mediate electron transfer.^35,36^ Given this, we tested whether **Eu** ^**III**^ **(LBC)** could be reduced by NADH in the presence of an enzyme moiety, *Hydrogenophilus thermoluteolus* (HoxFU), which is able to catalyze NADH oxidation and release two electrons to an iron sulfur electron relay chain.^37^ When **Eu** ^**III**^ **(LBC)** was gently stirred in aqueous solution (pH 7.4) containing excess NADH and a catalytic quantity of HoxFU under N_2_ for 12 h, *T*_1_ was measured to be 0.51 s, compared to 3.35 s in the absence of the enzyme. From the equation of the fit of *r*_1_ in Figure 2, it was estimated that 55% of **Eu** ^**III**^ **(LBC)** had been converted to **Eu** ^**II**^ **(LBC)** by HoxFU/NADH. The experimental value of 55% conversion of **Eu** ^**III**^ **(LBC)** to **Eu** ^**II**^ **(LBC)** is approaching the Nernstian equilibrium between NADH and the Eu complex, which predicts 65% conversion (see the Nernstian Calculation in the SI), indicating very efficient electron transfer from NADH to **Eu** ^**III**^ **(LBC)** via HoxFU.

HoxFU has high homology to the NADH-oxidizing moiety of mammalian respiratory Complex I.^38^ Using the methodology previously described, two other enzymes with an iron–sulfur cluster relay chain, [NiFe] hydrogenase-1 (Hyd-1) and hydrogenease-2 (Hyd-2) from *Escherichia coli*, were also found to reduce the complex under a H_2_ atmosphere. We then tested two flavoenzymes, plant ferredoxin-NADP^+^ reductase (FNR) and human cytochrome P450 reductase (POR); both showed low-level activity for NADPH-dependent reduction of **Eu** ^**III**^ **(LBC)**. Reduced equine cytochrome c (cyt c_red_) gave no detectable reduction of the complex, which is consistent with its relatively high midpoint potential, +0.25 V.^39^ Reduced flavins, FMNH_2_ and FADH_2_, also gave insignificant conversion (Figure 3).

**Figure 3:**
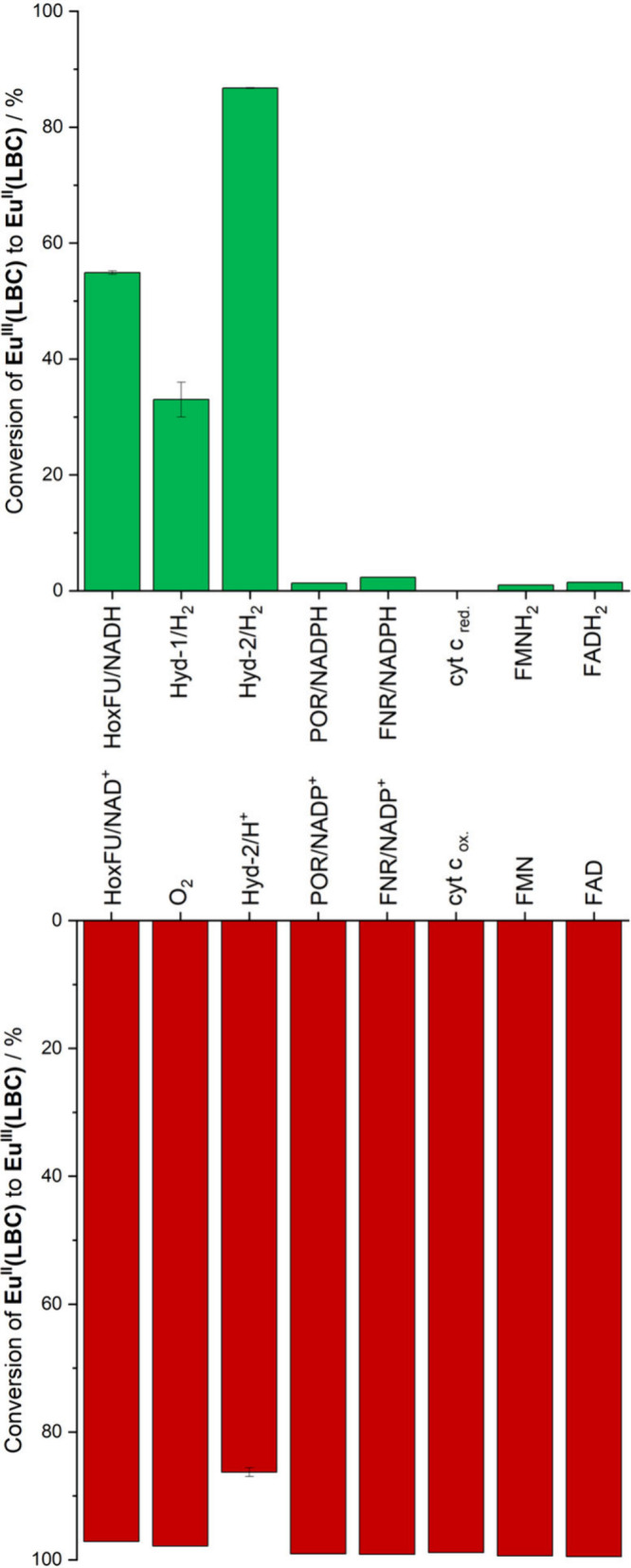
Estimated conversion of **Eu** ^**III**^ **(LBC)** to **Eu** ^**II**^ **(LBC)** by reductase systems and redox-active biomolecules at pH 7.4 after 12 h estimated from the equation of the linear fit of *r*_1_ for **Eu** ^**II**^ **(LBC)**, and below the estimated conversion of **Eu** ^**II**^ **(LBC)** to **Eu** ^**III**^ **(LBC)** by such systems.

Having explored enzyme-mediated reduction of **Eu** ^**III**^ **(LBC)**, we then explored the reverse reactions (see Tables S14 and S16–S21). Treatment of **Eu** ^**II**^ **(LBC)** with HoxFU and NAD^+^ showed clear evidence for a change in *T*_1_ consistent with near-complete oxidation to **Eu** ^**III**^ **(LBC)**. Similar oxidation to **Eu** ^**III**^ **(LBC)** was observed with FNR/NADP^+^ and POR/NADP^+^ and with the reversible hydrogenase, Hyd-2 under N_2_, where the enzyme reduces H^+^. In the case of Hyd-2, initial reduction of **Eu** ^**III**^ **(LBC)** under a H_2_ atmosphere was almost fully reversed when the sample was placed under a N_2_ atmosphere. Importantly, this indicates that the redox behavior of the **Eu­(LBC)** complex is switchable, depending on the redox environment.

Moreover, **Eu** ^**II**^ **(LBC)** is also oxidized by cyt c_ox_, as judged both by changes in *T*_1_ of bulk H_2_O and by the growth of absorption bands at 520 and 550 nm associated with cyt c_red_ (Figure S13).^40^ Simply aerating **Eu** ^**II**^ **(LBC)** also resulted in essentially full conversion to **Eu** ^**III**^ **(LBC)** (Table S15) confirming the important role O_2_ can have in oxidation of the system. Oxidized flavins, FAD and FMN, were also able to oxidize **Eu** ^**II**^ **(LBC)** (Figure 4).

**Figure 4:**
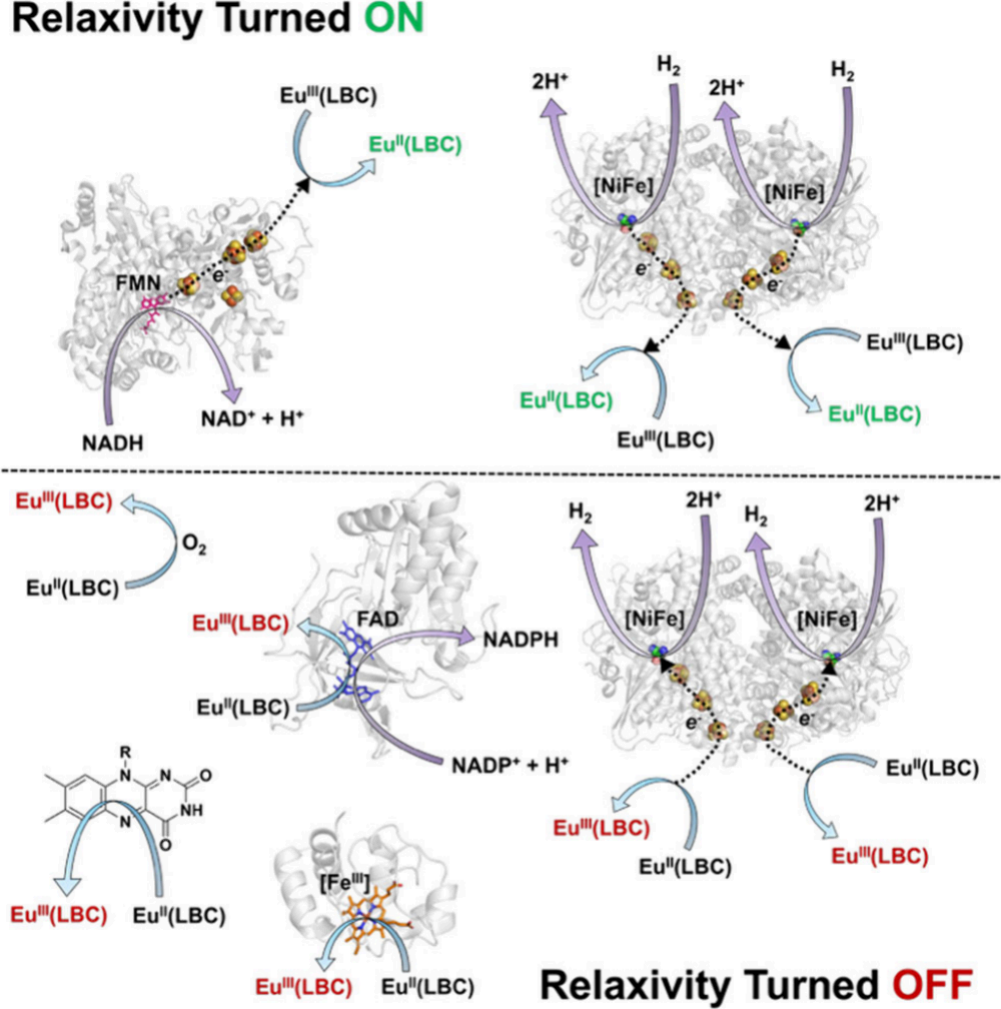
Depiction of NADH oxidation occurring at the FMN active site of HoxFU^41^ and subsequent electron transfer along the [Fe–S] cluster chain to reduce **Eu** ^**III**^ **(LBC)** to **Eu** ^**II**^ **(LBC)**; depiction of H_2_ oxidation occurring at the [NiFe] active site of Hyd-2 ^42^ and subsequent electron transfer along the [Fe–S] cluster chain to reduce **Eu** ^**III**^ **(LBC)** to **Eu** ^**II**^ **(LBC)** and the reverse reaction with electron transfer from **Eu** ^**II**^ **(LBC)** to the [Fe–S] cluster chain and back along the chain to the [NiFe] active site where protons are reduced to form H_2_, resulting in regeneration of **Eu** ^**III**^ **(LBC)**; depiction of oxidation of **Eu** ^**II**^ **(LBC)** to **Eu** ^**III**^ **(LBC)** at FAD in FNR^43^ and subsequent electron transfer to NADP^+^ to form NADPH; depiction of oxidation of **Eu** ^**II**^ **(LBC)** to **Eu** ^**III**^ **(LBC)** by cyt c_ox_ ^44^ at the [Fe^III^] heme site; depiction of oxidation of **Eu** ^**II**^ **(LBC)** to **Eu** ^**III**^ **(LBC)** by O_2_; and depiction of oxidation of **Eu** ^**II**^ **(LBC)** to **Eu** ^**III**^ **(LBC)** by flavins FMN and FAD.

In conclusion, we have shown that **Eu­(LBC)** has a reversible Eu^III/II^ redox couple that can be modulated by a series of biological oxidizing and reducing agents. The midpoint potential of this couple falls within a window which would be relevant to the reducing environment in hypoxic cells. We have demonstrated that **Eu** ^**III**^ **(LBC)** has negligible relaxivity whereas **Eu** ^**II**^ **(LBC)** has high relaxivity, comparable with commercial contrast agents such as **Gd** ^**III**^ **(DOTA)** (Dotarem). The reversibility of the **Eu** ^**III/II**^ **(LBC)** system in the context of biological redox processes thus results in switchable relaxivity. Such dynamic behavior is potentially of great relevance to providing clinical contrast media that respond to hypoxic environments. Furthermore, these properties can potentially be combined with the luminescence properties of **Eu** ^**III**^ **(LBC)** to achieve bimodal imaging. We are currently exploring the potential of this system for translation to such applications.

## Supplementary Material



## Notes

Additional experimental details, synthesis and characterization, electrochemical characterization and reduction assays (PDF)

## §

Department of Chemistry, Imperial College London, Molecular Sciences Research Hub, 82 Wood Lane, White City Campus, London W12 0BZ, United Kingdom

## ∇

Department of Chemistry, King’s College London, London SE1 1DB, United Kingdom.
