# Supplementary material for: Reversible Enzymatic Switching of the Oxidation State of a EuIII/II Complex Controls Relaxivity
Source: J Am Chem Soc. 2025 Oct 16;147(43):38992–7. doi: 10.1021/jacs.5c13447 (PMC12576775; doi:10.1021/jacs.5c13447)
Supplement: Supplementary file 1 [file ja5c13447_si_001.pdf]

Supporting Information for

Reversible Enzymatic Switching of the Oxidation State of a $Eu^{III/II}$ Complex Controls Relaxivity

Euan T. Sarson[a], Saul M. Cooper[a], Adam C. Sedgwick[a], Amy-Grace Berger[a], Sophie A. Twigger[b], Ester M. Hammond[b], Kylie A. Vincent[a]* and Stephen Faulkner[a]*

[a] Department of Chemistry, Chemistry Research Laboratory, University of Oxford, Mansfield Road, Oxford, OX1 3TA, United Kingdom

[b] Department of Oncology, Old Road Campus Research Building, University of Oxford, Oxford, OX3 7DQ

* Correspondence: kylie.vincent@chem.ox.ac.uk, stephen.faulkner@chem.ox.ac.uk

Contents

1. General Methods and Reagents ... 3
2. Enzymes ... 4
3. Characterisation Information ... 5
4. Abbreviations ... 6
5. Synthesis ... 7
5.1 Synthesis of **Eu^{III}(DOTA)** ... 7
5.2 Synthesis of **Eu^{III}(TAC)** ... 8
5.3 Synthesis of **Eu^{III}(DTDCC)** ... 10
5.4 Synthesis of **Eu^{III}(TPC)** ... 14
5.5 Synthesis of **Eu^{III}(LBC)** ... 16
5.6 Synthesis of **Eu^{II}(LBC)** ... 19
5.7 Synthesis of **Eu^{II}(221)** ... 20
5.8 Synthesis of **Eu^{II}(222)** ... 21
5.9 Synthesis of **Eu^{II}(222B)** ... 22
6. Cyclic Voltammetry ... 23
7. T_1 and Relaxivity Measurements ... 30
8. Enzyme/Cofactor Assays ... 32
9. Nernstian Calculation ... 37
10. NMR Spectra ... 38
References ... 43

1. General Methods and Reagents

Unless stated otherwise, experiments were performed at 25 °C using reagents and solvents purchased commercially and used without further purification. Anhydrous solvents were acquired by passing them through an MBraun MPSP-800 column followed by degassing with nitrogen. Deionised, microfiltered water was obtained from a Milli-Q™ Millipore machine. Thin layer chromatography was performed on silica-coated (60G F254) aluminium plates from Merck and aluminium oxide coated with 254 nm fluorescent indicator aluminium plates from Merck. Samples were visualized by UV-light (254 and 365 nm) and/or using permanganate stain.

Float-A-Lyzer® G2 dialysis tubes (500 MWCO) equipped with regenerated cellulose were purchased from Spectrum and used to purify the europium complexes. The dialysis tube was activated by 15% ethanol aqueous solution followed by MilliQ type 1 deionised water before being used. The corresponding complexes were dissolved in water and transferred into a dialysis tube. The dialysis tube was placed in a 2.5 L-beaker filled with MilliQ type 1 deionised water. The dialysis lasts for at least two days under stirring and the MilliQ water was replaced with fresh MilliQ water more than three times during dialysis.

2. Enzymes

The diaphorase moiety of the soluble hydrogenase from *Hydrogenophilus thermoluteolus* (HoxFU) was expressed heterologously in *Escherichia coli* according to a reported protocol,[1] and purified to a concentration of 12.4 mg/mL (M.W. 82000 Da).

dTM Hydrogenase-1 (Hyd-1) was produced in *E. coli* according to a reported protocol,[2] and purified to a concentration of 9.8 mg/mL (M.W. 94740 Da).

Hydrogenase-2 (Hyd-2) was produced in *E. coli* according to a reported protocol,[3] and purified to a concentration of 5.0 mg/mL (M.W. 94000 Da).

Cytochrome P450 reductase (POR) was purchased from BioIVT (formerly Cypex) - 'Human NADPH-CYP reductase expressed in *Escherichia coli*'; Catalogue Number: CYP004; Storage Buffer: 50 mM Tris-acetate (pH 7.6), 250 mM sucrose, 0.25 mM EDTA; Protein Concentration: 48.1 mg/mL (M.W. 78500 Da).

Ferredoxin $NADP^+$ reductase (FNR) was expressed in *E. coli* according to a reported protocol,[4] and purified to a concentration of 9.4 mg/mL (M.W. 45000 Da).

Cytochrome *c* from bovine heart (cyt *c*) was purchased from Merck (Sigma Aldrich) – 'Cytochrome *c* from bovine heart'; Product Number: C2037; solid powder (M.W. 12384 Da).

3. Characterisation Information

Low resolution mass spectra (LRMS) were carried out on a Waters LCT Premier XE bench-top orthogonal acceleration time-of-flight LC-MS system (without front end LC in direct infusion (loop injection)) connected to a Waters 1525u Binary HPLC Pump and a Waters/CTC Analytics 2777C Sample Manager. High resolution mass spectra (HRMS) were recorded on a BioAccord Waters RDa bench-top TOF used with an Acquity LC system in direct infusion (loop injection) mode for target confirmation.

NMR spectra were obtained using a Bruker Avance III HD nanobay NMR spectrometer equipped with a 9.4 T magnet (^{1}H 400.2 MHz, ^{13}C 100.6 MHz). Chemical shifts were referenced to residual solvent peaks and are given as follows: chemical shift (δ, ppm), multiplicity (s, singlet; br, broad; d, doublet, t, triplet; q, quartet; m, multiplet), coupling constant (*J*, Hz), integration. All NMR spectra were recorded at 298 K, unless stated otherwise.

UV-Vis absorbance spectra were recorded on an Agilent Cary 60 UV-Vis spectrometer using the Cary WinUV software Scan.

Cyclic Voltammetry (CV) was performed either in a small-volume electrochemical cell consisting of a Delrin cylinder containing a working electrode of glassy carbon (4 mm diameter), a counter electrode of graphite, and a leak-free Ag/AgCl reference electrode (LF-2-45 model from Alvatek Ltd) – Figures S6-13; or in an electrochemical cell consisting of a working electrode of glassy carbon (3 mm diameter), a Pt wire counter electrode, and a SCE reference electrode – Figures S14-15. The cell was set up under a N_2 atmosphere in a glove box (O_2 < 2 ppm), using 400 µL of a 1 mM solution of complex dissolved in degassed H_2O containing 50 mM Tris and 50 mM NaCl at pH 7.4. The air-tight electrochemical cell was then connected to an AutoLab 128 N potentiostat (Metrohm) controlled by Nova 2.1.7 software. Cyclic voltammograms were run with step potential = -0.00244 V and scan rate = 0.02 V/s, with 3 cycles recorded for each. The reference electrodes were calibrated to vs SHE by measuring the cyclic voltammogram of FcMeOH (0.1 mM in 4:1 buffer:EtOH) and comparing to a literature $E_{1/2}$ value of +420 mV vs SHE for FcMeOH.[5]

T_1 measurements were obtained using a Bruker Avance III NMR spectrometer equipped with a 11.75 T magnet (^{1}H 499.9 MHz) and using an inversion recovery pulse sequence. All T_1 measurements were recorded at 298 K. All degassed samples (100 µL) were placed in melting point tubes in a glove box under a N_2 atmosphere (O_2 < 2 ppm) which were then placed within Young's Tap NMR tubes and subsequently removed from the glove box for measuring. The acquired data was analysed using Bruker TopSpin.

4. Abbreviations

THF: tetrahydrofuran, **DCM**: dichloromethane, **DMF**: Dimethylformamide, **Et_2O**; Diethyl ether, **EtOAc**: Ethyl acetate, **MeCN:** acetonitrile, **TFA:** trifluoroacetic acid, **EtOH:** ethanol, **MeOH:** methanol, ***^{i}*PrOH**: Isopropanol, **rt:** room temperature; **MQ**: Milli-Q water.

5. Synthesis

5.1 Synthesis of **Eu^{III}(DOTA)**

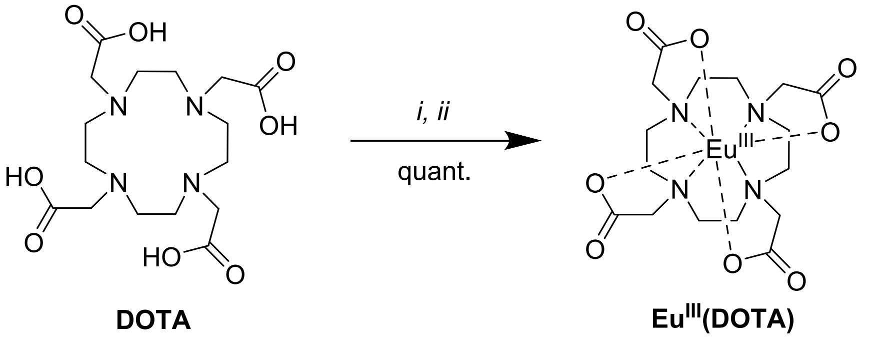

Scheme S1: The synthesis of **Eu^{III}(DOTA)** *Reagents and conditions: i)* Eu(OTf)$_3$, EtOH:H_2O (1:1), 40 ^oC, 12 h *ii)* NaOH, r.t., 2 h

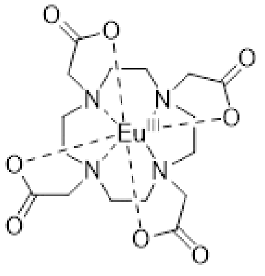

2,2′,2″,2‴-(1,4,7,10-Tetraazacyclododecane-1,4,7,10-tetrayl)tetraacetic acid (**DOTA**) (67.1 mg, 166 µmol) was added to Eu(OTf)$_3$ (119.8 mg, 200 µmol), followed by H_2O (0.95 mL) and EtOH (0.95 mL). The resulting solution was stirred at 40 ^oC for 12 h. Aqueous 1 M NaOH was added dropwise, adding 0.25 equiv every half hour to adjust to pH 7. The mixture was left stirring for a further 12 h, then centrifuged, the supernatant removed and the solvent of the supernatant was evaporated under reduced pressure. The crude product was redissolved in MQ water (7 mL) and purified by dialysis (10 mL dialysis tube, molecular weight cut-off: 500 Da). After 48 h and 6 MQ changes (4 L), the solvent was removed under reduced pressure to afford the desired complex **Eu^{III}(DOTA)** as a white crystalline powder (91.0 mg, quant). ^1H NMR (400.2 MHz, D_2O) δ (ppm): 33.75, 13.36, -0.76, -1.05, -2.08, -4.20, -6.66, -7.16, -7.97, -9.40, -14.54, -16.00; ^1H NMR in agreement with the literature.[6]

5.2 Synthesis of **Eu^{III}(TAC)**

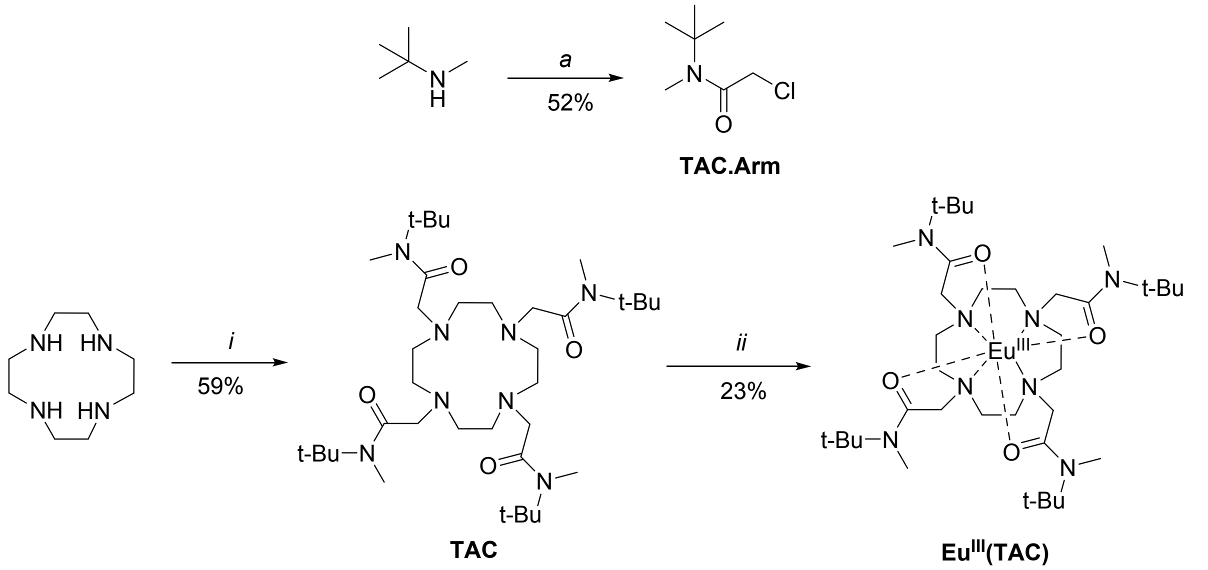

Scheme S2: The synthesis of **Eu^{III}(TAC)**. *Reagents and conditions: a)* chloroacetyl chloride, K_2CO_3, EtOAc:H_2O (3:2), r.t., 2 h *i)* **TAC.Arm**, Na_2CO_3, MeCN, 80 °C, 2 days *ii)* Eu(OTf)$_3$, EtOH:H_2O (1:1), 60 °C. 6 days

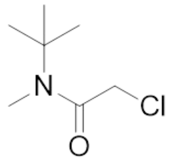

N-tert-butylmethylamine (1.38 mL, 11.5 mmol) and K_2CO_3 (4.75 g, 34.3 mmol) were dissolved in ethyl acetate (6 mL) and H_2O (4 mL.) Chloroacetyl chloride (1.10 mL, 13.8 mmol) was added dropwise with stirring. The reaction mixture was stirred for 2 h. EtOAc (20 mL) was added, and the organic layer was washed with H_2O (2 x 20 mL) and brine (20 mL) before being dried over anhydrous $MgSO_4$. The resulting solid was removed by filtering to obtain a pale-yellow solution; the solvent was removed under reduced pressure. $CHCl_3$ (10 mL) was added to the resulting crude oil and evaporated under reduced pressure to remove the EtOAc impurity, yielding N-methyl-N-tert-butylchloroacetamide as a pure yellow oil (**TAC.Arm**) (0.98 g, 52.1 %). ^{1}H NMR (400.2 MHz, $CDCl_3$) δ(ppm): 4.06 (s, 2H), 2.97 (s, 3H), 1.43 (s, 9H). ^{13}C NMR (100.6 MHz, $CDCl_3$) δ(ppm): 166.79, 57.51, 44.50, 32.41, 27.85. HRMS $[M + H]^+$ expected 164.08 ; found, 164.14.

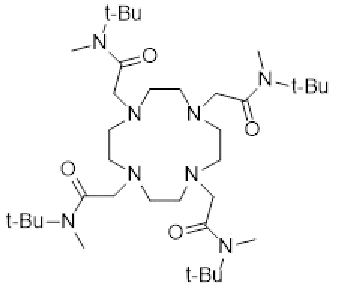

2,2',2'',2'''-(1,4,7,10-tetraazacyclododecane-1,4,7,10-tetrayl)tetrakis(*N*-(*tert*-butyl)-*N*-methylacetamide) (**TAC**) is a novel compound. **TAC.Arm** (0.24 g, 1.45 mmol) was dissolved in MeCN (5 mL). 1,4,7,10-tetraazacyclododecan (cyclen) (50 mg, 0.29 mmol) and Na_2CO_3 (0.31 g, 2.90 mmol) was added. This reaction mixture was heated under reflux at 80 °C with stirring. After 21.5 hours another equivalent of **TAC.Arm** was added (0.05 g, 0.29 mmol). After 25.5 hours another equivalent of **TAC.Arm** was added (0.05 g, 0.29 mmol). The reaction was stopped

after 44.5 hours. The reaction mixture was cooled to room temperature, filtered under gravity and concentrated under reduced pressure to afford a crude product (yellow solid). The crude product was purified via silica chromatography (100% hexane to 100% EtOAc to DCM/MeOH (80:20)) to give a white solid (0.12 g, 59.1 %). ^{1}H NMR (400.2 MHz, $CDCl_3$) δ(ppm): 3.60 (m, 3H), 3.50 (s, 8H), 3.02 (broad s, 4H), 2.77 (s, 12H), 2.13 (broad s, 7H), 1.81 (broad s, 2H), 1.37 (s, 36H). ^{13}C NMR (100.6 MHz, $CDCl_3$) δ(ppm): 171.17, 57.70, 56.98, 48.95. HRMS $[M + H]^+$ expected 682.02 ; found, 682.53

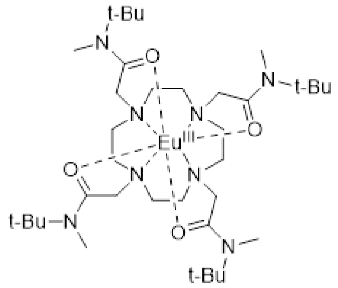

Eu^{III}(TAC) is a novel compound. **TAC** (50 mg) and $Eu(OTf)_3$ (1.1 equivalents) were dissolved in EtOH:H_2O 1:1 (2 mL) and stirred at 60 °C for 6 days. Solutions were neutralised with 1M NaOH. The solvent was removed under reduced pressure to give a crystalline white solid, which was purified by dialysis to give a yield of 0.021 g (22.6%). ^{1}H NMR (400.2 MHz, D_2O) δ(ppm): 38.93, 16.81, 14.63 – 13.46, 3.61, 3.31, 2.56 – -0.21, -2.02, -3.99, -5.91, -7.49, -9.35, -15.62, -16.53. LRMS: m/z calc. for $[M+OTf]^{2+}$ 491.22, found 491.14; $[M+2OTf]^+$ 1131.39, found 1131.34; $[M+2Cl]^+$ 904.40, found 904.34.

5.3 Synthesis of $\mathbf{Eu^{III}}$(DTDCC)

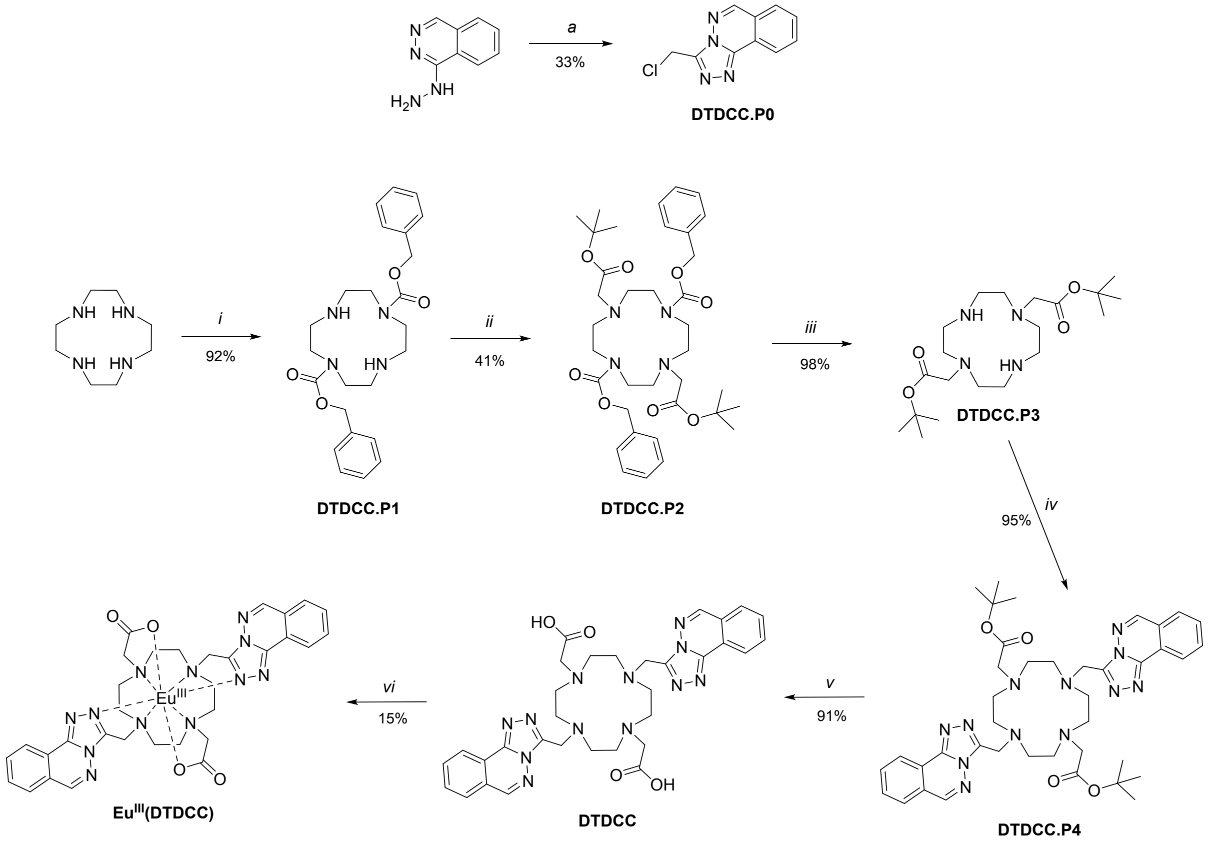

Scheme S3: The synthesis of $\mathbf{Eu^{III}}$**(DTDCC)**. *Reagents and conditions: a)* hydralazine hydrochloride, chloroacetyl chloride, $NaHCO_3$, H_2O, 110 °C, 16 h *i)* cyclen, benzyl chloroformate, $CHCl_3$, 0 °C → r.t., 16 h, N_2 *ii)* tert-butyl bromoacetate, K_2CO_3, dry MeCN, 0 °C → r.t., 16 h, N_2 *iii)* H_2, Pd/C, EtOH, r.t., 48 h *iv)* **DTDCC.P0**, K_2CO_3, dry MeCN, 92 °C, 48 h *v)* trifluoroacetic acid, DCM, r.t., 72 h *vi)* $Eu(OTf)_3$, MeOH, 60 °C, 7 days

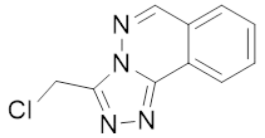

3-chloromethyl triazolo[3,4-α]phthalazine (**DTDCC.P0**) was synthesised by modifying a reported procedure.[7] Hydralazine hydrochloride (1.00 g, 5.08 mmol) and $NaHCO_3$ (0.44 g, 5.20 mmol) were dissolved in water (10 mL) to form a yellow solution. Chloroacetyl chloride (0.5 mL, 5.20 mmol) was then added dropwise to the solution to produce a colourless solution, which was then heated under reflux for 16 h at 110 °C. The white precipitate formed was collected by filtration and recrystallised from hot EtOH to produce pale yellow needles (0.36 g, 33%). ^{1}H NMR (400.2 MHz, $CDCl_3$) δ(ppm): 8.74 (aryl, s, 1H), 8.71 (aryl, d, 1H), 8.01 (aryl, m, 2H), 7.87 (aryl, m, 1H), 5.17 (CH_2Cl, s, 1H). ^{13}C NMR (100.6 MHz, $CDCl_3$) δ(ppm): 148.31, 147.32, 143.69, 134.47, 131.42, 128.39, 123.53, 123.36, 32.84. LRMS: m/z calc. for $[M+H]^+$ 219.04, found 219.0.

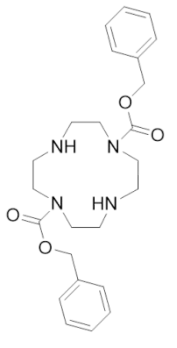

1,4,7,10-tetraaza-cyclododecane-1,7-dicarboxylic acid dibenzyl ester (**DTDCC.P1**) was synthesised by modifying a reported procedure.[8] A solution of cyclen (2.00 g, 11.6 mmol) in $CHCl_3$ (80 mL) was cooled on ice. Benzyl chloroformate (3.96 g, 23.2 mmol) was then added dropwise. A white solid was produced and the reaction mixture then left stirring under N_2 for 16 h at room temperature. The solvent was removed under reduced pressure and Et_2O added to the remaining solid. This was washed several times to give the product as the white dihydrochloride salt. The free base was obtained by addition of NaOH (150 mL, 2 M) to the solid and extraction of the aqueous phase with $CHCl_3$ (4 x 50 mL). The extracts were combined and dried over K_2CO_3. The solvent was removed to give the product as a clear oil (4.69 g, 92%). ^{1}H NMR (400.2 MHz, $CDCl_3$) *δ*(ppm): 7.38-7.27 (aryl, m, 10H), 5.14 (C*H*$_2$Ph, s, 4H), 3.41 (m, 8H), 2.83 (m, 8H). ^{13}C NMR (100.6 MHz, $CDCl_3$): δ(ppm): 156.87, 136.85, 128.56, 128.0, 127.85, 67.11, 50.88, 50.80, 49.69, 48.28. LRMS; m/z calc. for $[M+H]^+$ at 441.25, found 441.3.

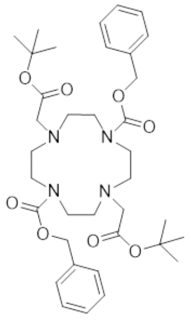

dibenzyl 4,10-bis(2-(tert-butoxy)-2-oxoethyl)-1,4,7,10-tetraazacyclododecane-1,7-dicarboxylate (**DTDCC.P2**) was synthesised by modifying a reported procedure.[8] **DTDCC.P1** (1.50 g, 3.41 mmol) was dissolved in anhydrous MeCN (50 mL) and K_2CO_3 (1.18 g, 8.51 mmol) added to the resulting solution. The reaction mixture was stirred for 20 min and cooled to 0 °C. Tert-butyl bromoacetate (1 mL, 6.83 mmol) was added dropwise over 30 min and the reaction was left stirring under N_2 for 16 h. The inorganic solid was filtered off and solvent removed under reduced pressure. The product was purified by silica gel chromatography (DCM/ MeOH 95:5 to 80:20 υ:υ) to give the final product as clear oil (1.23 g, 41%). ^{1}H NMR (400.2 MHz, $CDCl_3$) δ(ppm): 7.36-7.27 (aryl, m, 10H), 5.12 (C*H*$_2$Ph, s, 4H), 3.50-3.15 (m, 12H), 2.87 (s, 8H), 1.43 (s, 18H). ^{13}C NMR; (100 MHz, $CDCl_3$) δ(ppm): 170.70, 156.62, 137.0, 128.61, 128.06, 128.02, 81.10, 67.13, 56.16, 54.55, 54.25, 53.56, 47.20, 46.85, 28.33. LRMS; m/z calc for $[M+H]^+$ at 669.38, found at 669.35.

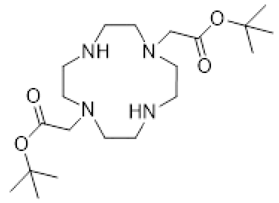

(7-tert-butoxycarbonylmethyl-1,4,7,10-tetraaza-cyclododec-1-yl)-acetic acid tert-butyl ester (**DTDCC.P3**) was synthesised by modifying a reported procedure.[8] **DTDCC.P2** (1.17 g, 1.75 mmol) was dissolved in a round bottom flask charged with Pd/C (0.20 g) and EtOH (20 mL). The suspension was bubbled through with H_2 for 5 min and then left stirring in a H_2 atmosphere for 48 h. The mixture was filtered over celite and the solvent removed under reduced pressure. The oil was dissolved in MeOH and precipitated by Et_2O. The supernatant was decanted off and the solid was dried under reduced pressure to give the

product as an off-white powder (0.69 g, 98%). ^{1}H NMR (400 MHz, $CDCl_3$) δ(ppm): 3.32 (s, 4H), 2.82 (s, 8H), 2.63 (t, 8H), 1.46 (s, 18H). LRMS: m/z calc. for $[M+H]^+$ at 401.30, found at 401.3.

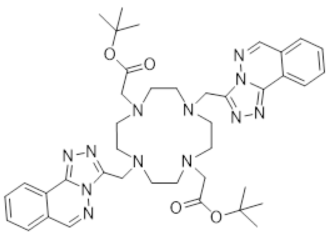

DTDCC.P4 is a novel compound. **DTDCC.P3** (0.53 g, 1.32 mmol) and K_2CO_3 (0.53 g, 3.80 mmol) were dissolved in anhydrous MeCN (50 mL). 2 equivalents of compound 1.2 (0.58 g, 2.64 mmol) were then added to the reaction mixture and the resulting yellow suspension left stirring under reflux for 48 h at 92 °C. The inorganic solid was filtered off and the solvent removed under reduced pressure to give a pale orange solid. The product was purified by alumina column chromatography (DCM:MeOH, 95:5 to 90:10 v:v) to give the final product (0.96 g, 95%). ^{1}H NMR (400.2 MHz, $CDCl_3$) δ(ppm): 8.82 (aryl, s, 2H), 8.55 (aryl, d, J = 7.9, 2H), 8.05 (aryl, d, J = 7.9, 2H), 7.97 (aryl, m, 2H), 7.86 (aryl, m, 2H), 4.23 (s, 4H), 3.3–2.38 (m, 12H), 1.30 (s, 18H). LRMS; m/z calc. for $[M+2H]^{2+}$ at 383.22, found at 383.3. IR (solid); v_{max} (cm^{-1}) = 3384, 2780, 2829, 2225, 2162, 2035, 1980, 1721, 1626, 1527.

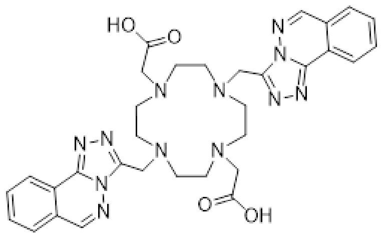

DTDCC is a novel compound. **DTDCC.P4** (0.96 g, 1.25 mmol) was dissolved in DCM (4 mL). Trifluoroacectic acid (9 mL) was then added to the solution and the reaction mixture left to stir for 72 h. The solvents were then removed under reduced pressure and the orange oil washed several times with MeOH (4 x 5 mL) to yield an orange solid as product (0.74 g, 91%). ^{1}H NMR (400 MHz, D_2O) δ(ppm): 9.00 (aryl, s, 2H), 8.15 (aryl, d, J = 8, 2H), 7.90 (aryl, m, 2H), 7.69 (aryl, m, 4H), 4.55 (CH_2COOH, s, 4H), 4.23 (CH_2Ar, s, 4H), 3.93-3.67 (CH_2N ring, m, 8H), 3.30 (CH_2N ring, m, 8H). LRMS; m/z calc. for $[M+H]^+$ at 653.30, found at 653.3. IR (solid); v_{max} (cm^{-1}) = 3092, 2917, 2849, 2541, 1725, 1676, 1540. UV-Vis (H_2O); λ_{max} (nm) = 240, 247, 264.

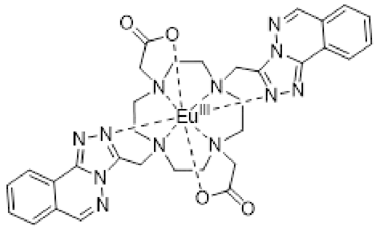

Eu^{III}(DTDCC) is a novel compound. To a solution of **DTDCC** (100 mg, 0.307 mmol) in methanol (2 mL), $Eu(OTf)_3$ was added (1.1 eq.), and the reaction mixture was stirred at 60 °C. After seven days, the reaction was stopped and the volatiles were removed under reduced pressure. The remaining solid was redissolved in minimal MeOH and precipitated by addition of Et_2O. After decantation of the supernatant, the solid was dried under vacuum. The product was dissolved in minimal H_2O and purified by dialysis over 48 h to yield the product as a white powder (18 mg, 15%). ^{1}H

NMR (400.2 MHz, D_2O) δ(ppm): 28.70, 23.20, 16.69, 12.29, 11.99, 10.52, 9.47, 9.08, 1.73, 0.43, -0.81, -7.04, -8.08, -11.09, -11.74, -13.84, -18.32, -22.79, -23.23, -23.70. LRMS; m/z calc. for $[M]^+$ at 803.20, found at 803.22.

5.4 Synthesis of **Eu^{III}(TPC)**

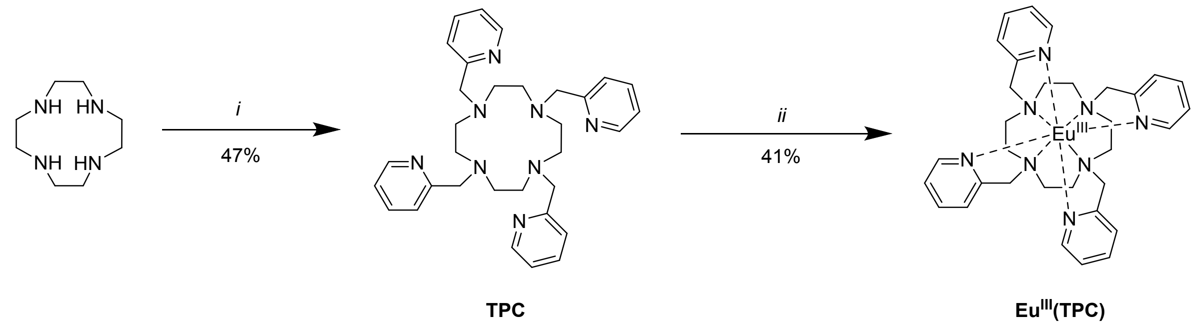

Scheme S4: The synthesis of **Eu^{III}(TPC)**. *Reagents and conditions: i)* Cs_2CO_3, 2-picolylchloride hydrochloride, MeCN, 82 °C, 24 h, Ar *ii)* $Eu(OTf)_3$, MeOH, 40 °C, 24 h

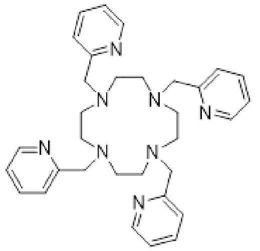

1,4,7,10-tetrakis(2-pyridylmethyl)-1,4,7,10-tetraazacyclododecane (**TPC**) was synthesised by modifying a reported procedure.[9] Cyclen (0.32 g, 1.88 mmol) was dissolved in MeCN (15 mL), and Cs_2CO_3 added (12.45 g, 38.21 mmol) to form a suspension. 2-picolylchloride hydrochloride (1.43 g, 8.71 mmol) was dissolved in MeCN (23 mL), and this solution was added dropwise to the suspension resulting in a colour change from colourless to pink to light brown. The resulting solution was stirred at 82 °C for 24 h under a N_2 atmosphere. The solution was filtered to remove the inorganic salts, and all volatiles were removed under reduced pressure to yield a crude powder. The powder was subsequently extracted into hot MeCN (50 mL), filtered, and cooled to -20 °C overnight. The resultant orange crystals were collected by filtration the next day, washed with minimal volumes of MeCN, and dried under vacuum to yield analytically pure **TPC** as an orange crystalline powder (472.3 mg, 47%). ^{1}H NMR (400.2 MHz, $CDCl_3$) δ (ppm): 8.45 (dd, 4H, $^3J_{HH}$ = 4.8 Hz, $^4J_{HH}$ = 0.9 Hz), 7.68 (d, 4H, $^3J_{HH}$ = 7.8 Hz), 7.40 (td, 4H, $^3J_{HH}$ = 7.6 Hz, $^4J_{HH}$ = 1.7 Hz), 7.06 (dd, 4H, $^3J_{HH}$ = 7.6 and 4.9 Hz), 3.61 (s, 8H), 2.75 (s, 16H); ^{13}C NMR (100.6 MHz, $CDCl_3$) δ (ppm): 160.5, 149.0, 136.3, 123.0, 121.8, 61.8, 53.6; LRMS(MeOH): 537.20 {M + H}$^+$ (75%), 559.21 {M + Na}$^+$ (100%) calc. for $C_{32}H_{40}N_8$, *M*r = 536.34. ^{1}H and ^{13}C NMR in agreement with the literature.[9]

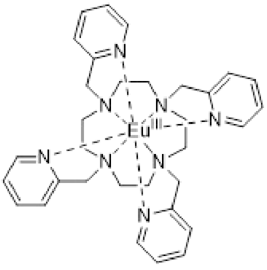

Eu^{III}(TPC) was synthesised by modifying a reported procedure.[9] A solution of **TPC** (101.0 mg, 188 μmol) in MeOH (3 mL) was added dropwise to $Eu(OTf)_3$ (123.0 mg, 205 μmol) in MeOH (2 mL) and stirred at 40 °C for 24 h. The reaction mixture was cooled to room temperature and the solvent removed under reduced pressure. The off-white powder was dissolved in MeCN (3 mL) which was equally divided between two vials. Et_2O (2 x 5 mL) was layered onto each solution respectively. After 3 days, diffusion was complete, and the precipitated white solid was isolated by filtration under reduced pressure

and washed with Et_2O to produce **Eu^{III}(TPC)** as a white crystalline solid (17.2 mg, 41%). ^1H NMR (400.2 MHz, D_2O) δ (ppm): major isomer with all peaks observed as broad singlets 9.73, 7.65, 6.36, 4.92, 4.24, 0.58, -0.09, -1.59, -13.21, -16.54; LRMS(MeOH): 986.98 $\{M - OTf\}^+$ (19%) calc. for $C_{35}H_{40}EuF_9N_8O_9S_3$, *M*r = 1136.11. ^1H NMR in agreement with the literature.[9]

5.5 Synthesis of **Eu^{III}(LBC)**

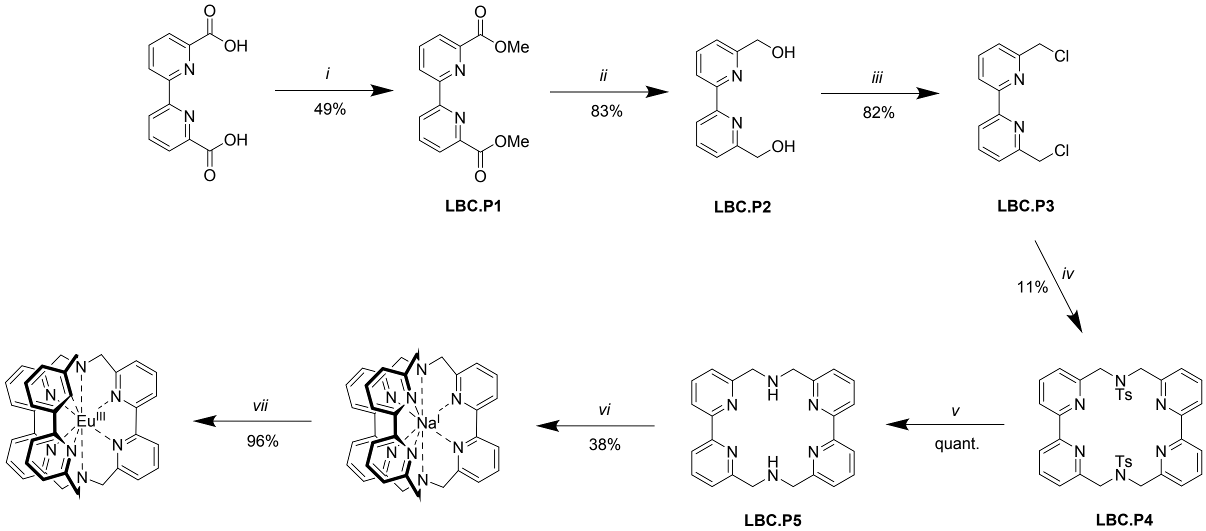

Scheme S5: The synthesis of **Eu^{III}(LBC)**. *Reagents and conditions: i)* K_2CO_3, MeI, DMF, 50 °C, 12 h *ii)* $NaBH_4$, MeOH:THF (2:1), r.t., 1 h *iii)* $SOCl_2$, DCM, r.t., 12 h, Ar *iv) p*-toluenesulfonamide monosodium salt, anhydrous EtOH, 90 °C, 24 h, Ar *v)* conc H_2SO_4, 110 °C, 2 h *vi)* Na_2CO_3, **LBC.P3**, anhydrous MeCN, 82 °C, 48 h, Ar *vii)* $Eu(OTf)_3$, anhydrous MeCN, 82 °C, 48 h, Ar

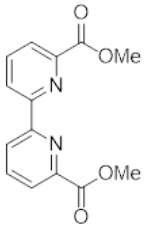

dimethyl 2,2'-bipyridine-6,6'-dicarboxylate (**LBC.P1**) was synthesised by modifying a reported procedure.[10] 2,2'-bipyridine-6,6'-dicarboxylic acid (2.94 g, 12.04 mmol), K_2CO_3 (7.39 g, 53.51 mmol), and MeI (1.65 mL, 2.20 equiv) were dissolved in DMF (60 mL) and stirred at 50 °C for 12 h. The reaction mixture was cooled to room temperature, divided into two equal parts and each part mixed with brine (270 mL), H_2O (30 mL) and EtOAc (300 mL). The organic layer was isolated, and the aqueous portion extracted with EtOAc (300 mL). The organic layers were combined and extracted with brine (100 mL). Combined organic extracts were dried with anhydrous $MgSO_4$ and filtered. The solvent was removed under reduced pressure to give **LBC.P1** as a yellow/white crystalline powder (1.59 g, 49%). ^{1}H NMR (400.2 MHz, $CDCl_3$) δ (ppm): 8.75 (dd, 2H, $^{3}J_{HH}$ = 7.9 Hz, $^{4}J_{HH}$ = 1.0 Hz), 8.16 (dd, 2H, $^{3}J_{HH}$ = 7.8 Hz, $^{4}J_{HH}$ = 1.1 Hz), 7.99 (t, 2H, $^{3}J_{HH}$ = 7.9 Hz), 4.03 (s, 6H); ^{13}C NMR (100.6 MHz, $CDCl_3$) δ (ppm): 165.8, 155.6, 147.7, 138.2, 125.6, 125.0, 53.0. ^{1}H and ^{13}C NMR in agreement with the literature.[11]

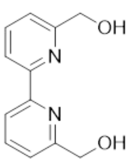

6,6'-bis(hydroxymethyl)-2,2'-bipyridine (**LBC.P2**) was synthesised by modifying a reported procedure.[10] **LBC.P1** (1.59 g, 5.84 mmol) was dissolved in MeOH (45 mL) and THF (26 mL). $NaBH_4$ (1.33 g, 35.21 mmol) was slowly added to the stirring mixture and the colour changed from yellow to pink. The reaction was left to stir for 1 h and turned colourless. The reaction mixture was cooled to room temperature and extracted with NH_4Cl (400 mL) and EtOAc (2 x 400 mL). Combined organic extracts were dried with anhydrous $MgSO_4$ and filtered. The solvent was removed under reduced pressure to give a colourless oil, and left to evaporate for 12 h in air to give crude **LBC.P2** as a white powder.

The crude powder was redissolved in a minimal amount of acetone and loaded onto a neutral alumina column. Elution with $CHCl_3$:acetone:MeOH (100:0:0 → 95:5:0 →…→ 80:20:0 → 80:19:1 → 80:18:2 →…→ 80:0:20) followed by removal of the solvent under reduced pressure afforded the product **LBC.P2** as a white powder (1.05 g, 83%). ^{1}H NMR (400.2 MHz, DMSO-*d*$_6$) δ (ppm): 8.24 (d, 2H, $^3J_{HH}$ = 7.8 Hz), 7.94 (t, 2H, $^3J_{HH}$ = 7.7 Hz), 7.53 (d, 2H, $^3J_{HH}$ = 7.7 Hz), 4.67 (s, 4H); ^{13}C NMR (100.6 MHz, DMSO-*d*$_6$) δ (ppm): 161.7, 154.2, 137.6, 120.5, 118.6, 64.4. ^{1}H and ^{13}C NMR in agreement with the literature.[12]

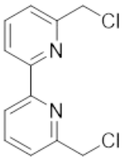

6,6'-di(chloromethyl)-2,2'-bipyridine (**LBC.P3**) was synthesised by modifying a reported procedure.[10] **LBC.P2** (1.05 g, 4.86 mmol) was dissolved in DCM (75 mL) and put under an Ar atmosphere. $SOCl_2$ (6.31 mL, 86.95 mmol) was slowly added to the stirring mixture to create a cloudy suspension and was left overnight. The reaction mixture was poured over ice (200 g) and stirred until the ice melted. The solution was neturalised with 2 M NaOH and extracted with DCM (3 x 100 mL). Combined organic extracts were extracted with H_2O (150 mL) and then brine (150 mL), dried with anhydrous $MgSO_4$ and filtered. The solvent was removed under reduced pressure to give **LBC.P3** as a white crystalline powder (1.01 g, 82%). ^{1}H NMR (400.2 MHz, $CDCl_3$) δ (ppm): 8.40 (dd, 2H, $^3J_{HH}$ = 8.0 Hz, $^4J_{HH}$ = 0.8 Hz), 7.85 (t, 2H, $^3J_{HH}$ = 7.8 Hz), 7.51 (d, 2H, $^3J_{HH}$ = 7.7 Hz, $^4J_{HH}$ = 0.9 Hz), 4.76 (s, 4H); ^{13}C NMR (100.6 MHz, DMSO-*d*$_6$) δ (ppm): 156.2, 155.5, 138.1, 123.0, 120.6, 47.1. ^{1}H and ^{13}C NMR in agreement with the literature.[12]

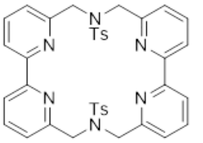

LBC.P4 was synthesised by modifying a reported procedure.[10,13] Na (0.50 g, 22 mmol) was added to EtOH (20 mL) while stirring and left for 20 min. The solvent was removed under reduced pressure to afford NaOEt as a white powder. Anhydrous EtOH (40 mL) was immediately added to the just synthesised NaOEt (1.64 g, 24.06 mmol) and *p*-toluenesulfonamide (4.10 g, 23.95 mmol) and was heated under reflux at 90 ^oC for 3 h. The reaction mixture was cooled to room temperature, filtered under reduced pressure and washed with EtOH to yield *p*-toluenesulfonamide monosodium salt as a white powder (2.96 g, 64%). **LBC.P3** (332.0 mg, 1.31 mmol) and *p*-toluenesulfonamide monosodium salt (507.0 mg, 2.62 mmol) were refluxed in anhydrous EtOH (40 mL) at 90 ^oC for 24 h. Initially, the reaction mixture went transparent and 12 h later a yellow-white precipitate had formed. The reaction mixture was cooled to -20 ^oC, filtered under reduced pressure and washed with H_2O and EtOH to afford **LBC.P4** as an insoluble off-white solid (0.19 g, 11%). This compound was taken forward without characterisation or further purification.

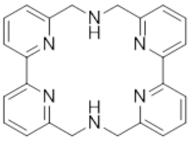

2,17-diaza[3.3](6,6')-2,2'-bipyridinophane (**LBC.P5**) was synthesised by modifying a reported procedure.[10,13] **LBC.P4** (0.19 g, 279.0 μmol) was dissolved in concentrated H_2SO_4 (3.57 mL) to form a gold coloured solution

and refluxed at 110 °C while stirring for 2 h. The reaction mixture was cooled to room temperature, H_2O (2.9 mL) was added and the pH of the solution was adjusted to pH 10 using 2M $NaOH_{(aq)}$ at which point a precipitate formed. The aqueous layer was extracted with $CHCl_3$ (3 x 30 mL) and combined organic extracts were dried with anhydrous $MgSO_4$ and filtered. The solvent was removed under reduced pressure to give **LBC.P5** as an insoluble white solid (110.1 mg, quant.). This compound was taken forward without characterisation or further purification.

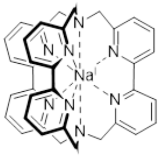

Na^{I}(LBC) was synthesised by modifying a reported procedure.[10,13] **LBC.P5** (75.0 mg, 190 µmol), **LBC.P3** (50.0 mg, 190 µmol) and Na_2CO_3 (162.0 mg, 1.53 mmol) were put under Ar and combined with dry MeCN (100 mL). The reaction mixture was refluxed at 82 °C for 48 h with stirring, cooled and filtered under reduced pressure. The solvent was removed under reduced pressure to give crude **Na^{I}(LBC)** as a white powder. The crude powder was redissolved in a minimal amount of $CHCl_3$ and loaded onto a silica gel column. Elution with $CHCl_3$:MeOH (100:0 → 99:1 →…→ 95:5 → 90:10 → 85:15 → 80:20) followed by removal of the solvent under reduced pressure afforded the product **Na^{I}(LBC)** as a white powder (46.0 g, 38%). ^{1}H NMR (400.2 MHz, $CDCl_3$) δ (ppm): 7.88 (d, 6H, $^{3}J_{HH}$ = 7.9 Hz, Ar H), 7.81 (t, 6H, $^{3}J_{HH}$ = 7.8 Hz, Ar H), 7.31 (d, 6H, $^{3}J_{HH}$ = 7.5 Hz, Ar H), 3.82 (s, 12H, CH_2); ^{13}C NMR (100.6 MHz, $CDCl_3$) δ (ppm): 158.6, 155.4, 138.3, 124.1, 120, 59.7; LRMS(MeOH): 597.083 {M} (100%) calc. for $C_{36}H_{30}N_8Na^+$, *M*r = 597.25. λ_{max} (H_2O)/nm 239 and 286 (values of ε unattainable because **Na^{I}(LBC)** is sparingly soluble in H_2O). ^{1}H and ^{13}C NMR in agreement with the literature.[13]

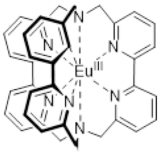

Eu^{III}(LBC) was synthesised by modifying a reported procedure.[10,14] **Na^{I}(LBC)** (24.6 mg, 38.9 µmol) and $Eu(OTf)_3$ (26.0 mg, 43.4 µmol) were put under Ar and dry MeCN (20 mL) was added. The reaction mixture was heated under reflux at 82 °C for 48 h, cooled to room temperature and the solvent removed under reduced pressure. The crude product was redissolved in minimum MeOH and Et_2O was added till a white ppt formed. This was left at -20 °C for 5 nights. The ppt was recovered by filtration and washed with Et_2O. This was redissolved in MQ (7 mL) and purified by dialysis (10 mL dialysis tube, molecular weight cut-off: 500 Da). After 7 days and 7 MQ changes (4 L), the solvent was removed under reduced pressure to afford the desired complex **Eu^{III}(LBC)** as a white powder (43.9 mg, 96%). ^{1}H NMR (400.2 MHz, D_2O) δ (ppm): 8.23 (s, 6H, Ar H), 7.66 (s, 6H, Ar H), 6.92 (s, 6H, Ar H), 0.31 (s, 12H, CH_2); LRMS(MeOH): 242.215 $\{M\}^{3+}$ (66%) calc. for $C_{37}H_{30}EuN_7$, *M*r = 725.18. λ_{max} (H_2O)/nm 245 and 303 (ε/dm^3 mol^{-1} cm^{-1} 17538 and 25000). ^{1}H NMR in agreement with the literature.[14]

5.6 Synthesis of **Eu^{II}(LBC)**

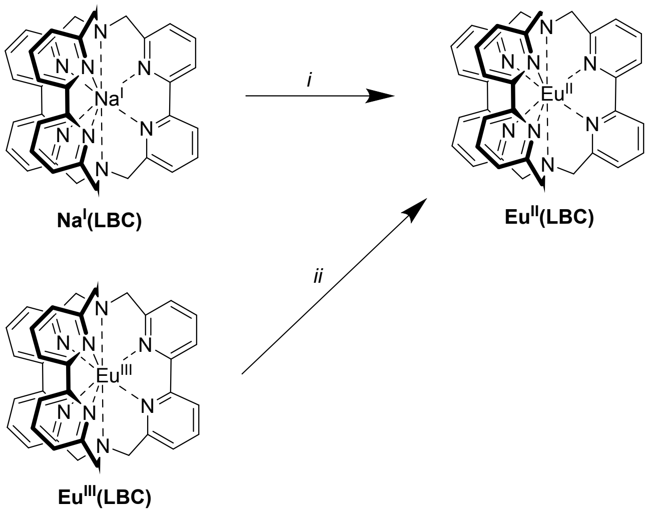

Scheme S6: The synthesis of **Eu^{II}(LBC)**. *Reagents and conditions: i)* $EuCl_2$, degassed H_2O (50 mM Tris, 50 mM NaCl, pH 7.4), r.t.,12 h, N_2 or *ii)* bulk electrolysis (chronoamperometry) by applying -0.610 V vs SHE for 12 h (50 mM NaCl)

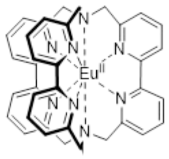

Eu^{II}(LBC) could be chemically synthesised from **Na^{I}(LBC)** or electrochemically synthesised from **Eu^{III}(LBC)**. In a glove box (O_2 < 2 ppm), **Na^{I}(LBC)** (1.90 mg, 3.00 μmol) and $EuCl_2$ (0.69 mg, 3.00 μmol) were combined and degassed H_2O (3 mL) was added and the reaction mixture was stirred at room temperature for 24 h to form a pink solution. Alternatively, an aqueous solution of **Eu^{III}(LBC)** (50 mM NaCl) was subjected to bulk electrolysis (chronoamperometry) by applying -0.610 V vs SHE for 12 h. λ_{max} (H_2O)/nm 242 and 295 (ε/dm^3 mol^{-1} cm^{-1} 24497 and 27266).

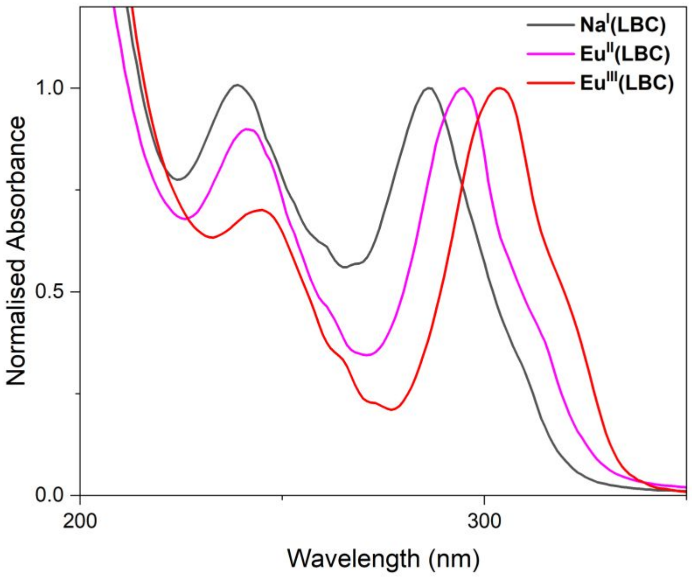

Figure S1: The normalised UV-Vis absorbance spectra of **Na^{I}(LBC)**, **Eu^{II}(LBC)**, and **Eu^{III}(LBC)** in degassed H_2O.

5.7 Synthesis of **Eu^{II}(221)**

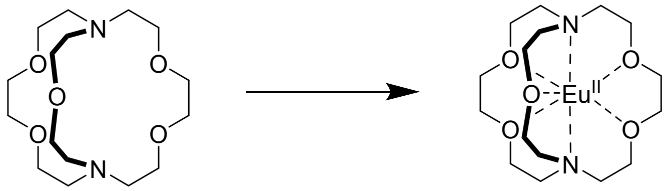

221 **Eu^{II}(221)**

Scheme S7: The synthesis of **Eu^{II}(221)**. *Reagents and conditions:* $EuCl_2$, degassed H_2O (50 mM Tris, 50 mM NaCl, pH 7.4), r.t.,12 h, N_2

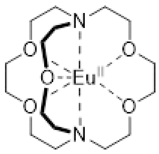

Eu^{II}(221) was synthesised by modifying a reported procedure.[15] In a glove box (O_2 < 2 ppm), 4,7,13,16,21-Pentaoxa-1,10-diazabicyclo[8.8.5]tricosane (**221**) (1.48 mg, 4.44 µmol) and $EuCl_2$ (0.89 mg, 4.00 µmol) were combined and of degassed H_2O (4 mL, 50 mM Tris, 50 mM NaCl, pH 7.4) was added. The reaction mixture was stirred at room temperature for 24 h then used directly for recording cyclic voltammetry.

5.8 Synthesis of **Eu^II^(222)**

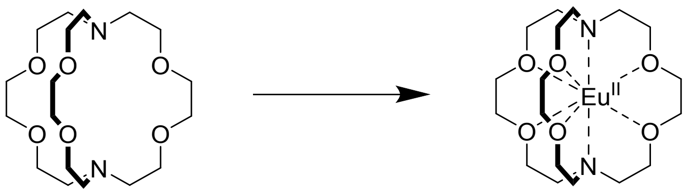

222 **Eu^{II}(222)**

Scheme S8: The synthesis of **Eu^{II}(222)**. *Reagents and conditions:* $EuCl_2$, degassed H_2O (50 mM Tris, 50 mM NaCl, pH 7.4), r.t.,12 h, N_2

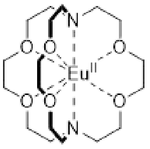

Eu^{II}(222) was synthesised by modifying a reported procedure.[15] In a glove box (O_2 < 2 ppm), 4,7,13,16,21,24-Hexaoxa-1,10-diazabicyclo[8.8.8]hexacosane (**222**) (1.67 mg, 4.44 µmol) and $EuCl_2$ (0.89 mg, 4.00 µmol) were combined and degassed H_2O (4 mL, 50 mM Tris, 50 mM NaCl, pH 7.4) was added. The reaction mixture was stirred at room temperature for 24 h then used directly for recording cyclic voltammetry.

5.9 Synthesis of **Eu^{II}(222B)**

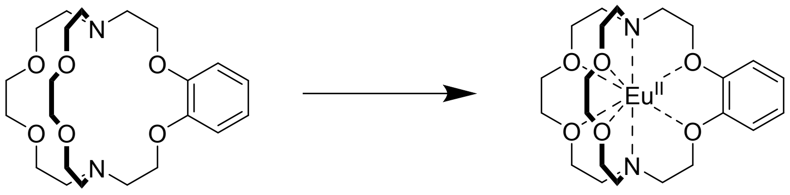

222B **Eu^{II}(222B)**

Scheme S9: The synthesis of **Eu^{II}(222B)**. *Reagents and conditions:* $EuCl_2$, degassed H_2O (50 mM Tris, 50 mM NaCl, pH 7.4), r.t.,12 h, N_2

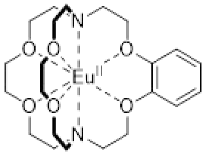

Eu^{II}(222B) was synthesised by modifying a reported procedure.[15] In a glove box (O_2 < 2 ppm), 5,6-Benzo-4,7,13,16,21,24-hexaoxa-1,10-diazabicyclo[8.8.8]hexacos-5-ene (**222B**) (1.88 mg, 4.44 µmol) and $EuCl_2$ (0.89 mg, 4.00 µmol) were combined and degassed H_2O (4 mL, 50 mM Tris, 50 mM NaCl, pH 7.4) was added. The reaction mixture was stirred at room temperature for 24 h then used directly for recording cyclic voltammetry.

6. Cyclic Voltammetry

Table S1: anodic, cathodic and midpoint potentials of various molecular Eu complexes in 50 mM Tris 50 mM NaCl H_2O at pH 7.4. Potentials are listed as ± standard error.

Sample	Anodic peak potential, E_{pa} vs SHE (V)	Cathodic peak potential, E_{pc} vs SHE (V)	Midpoint potential, $E_{1/2}$ vs SHE (V)
**Eu(DOTA)**	-0.776 ± 0.006	-1.046 ± 0.002	-0.911 ± 0.008
**Eu(TAC)**	-0.756 ± 0.001	-0.857 ± 0.002	-0.807 ± 0.002
**Eu(DTDCC)**	-0.588 ± 0.001	-0.673 ± 0.001	-0.631 ± 0.002
**Eu(TPC)**	-0.354 ± 0.001	-0.424 ± 0.002	-0.389 ± 0.002
**Eu(LBC)**	-0.302 ± 0.002	-0.418 ± 0.003	-0.360 ± 0.004
**Eu(221)**	-0.102 ± 0.003	-0.280 ± 0.009	-0.191 ± 0.007
**Eu(222)**	+0.073 ± 0.002	-0.010 ± 0.001	+0.032 ± 0.003
**Eu(222B)**	+0.362 ± 0.002	-0.085 ± 0.006	+0.139 ± 0.005

The second of three cycles of the CV is shown for each complex; the arrow indicates the initial direction of scan.

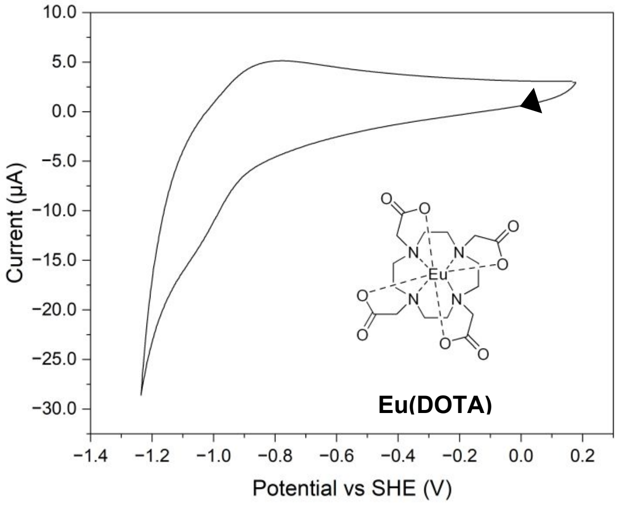

Figure S2: CV of **Eu(DOTA)** in 50 mM Tris 50 mM NaCl H_2O at pH 7.4, starting from **Eu^{III}(DOTA)**, with scan rate 0.02 V/s scanning cathodically.

The reduction and oxidation events of $Eu^{III/II}$ are not as defined for **Eu(DOTA)** because of overlap of the waves with the aqueous solvent window, as previously observed.[16]

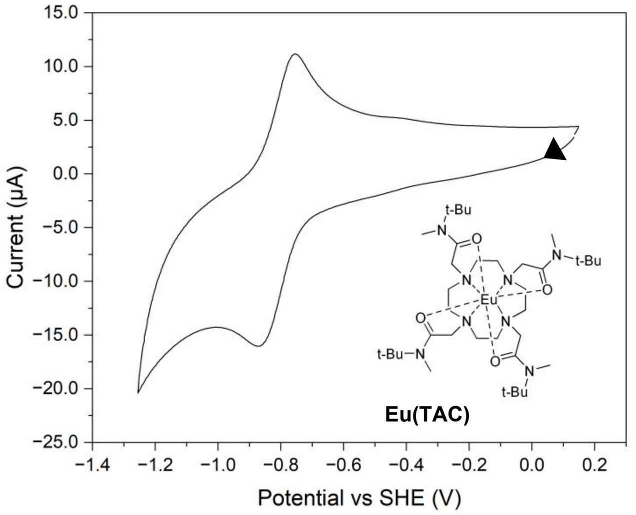

Figure S3: CV of **Eu(TAC)** in 50 mM Tris 50 mM NaCl H_2O at pH 7.4, starting from **Eu^{III}(TAC)**, with scan rate 0.02 V/s scanning cathodically.

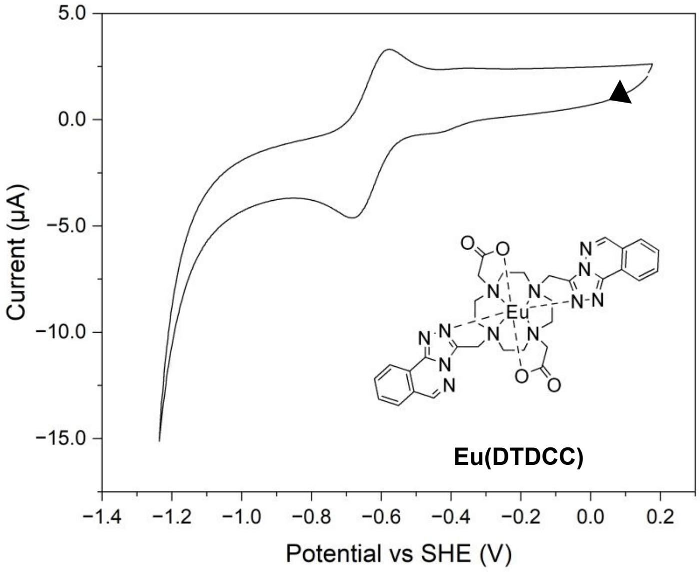

Figure S4: CV of **Eu(DTDCC)** in 50 mM Tris 50 mM NaCl H_2O at pH 7.4, starting from **Eu^{III}(DTDCC)**, with scan rate 0.02 V/s scanning cathodically.

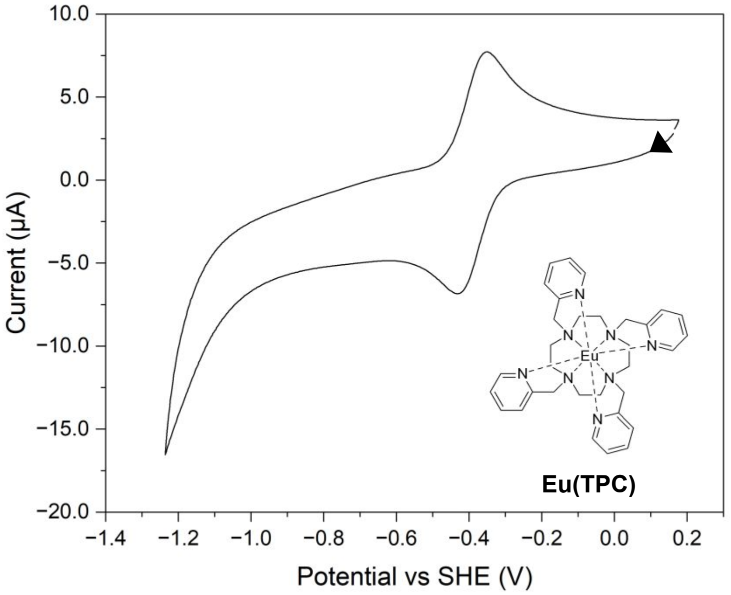

Figure S5: CV of **Eu(TPC)** in 50 mM Tris 50 mM NaCl H_2O at pH 7.4, starting from **Eu^{III}(TPC)**, with scan rate 0.02 V/s scanning cathodically.

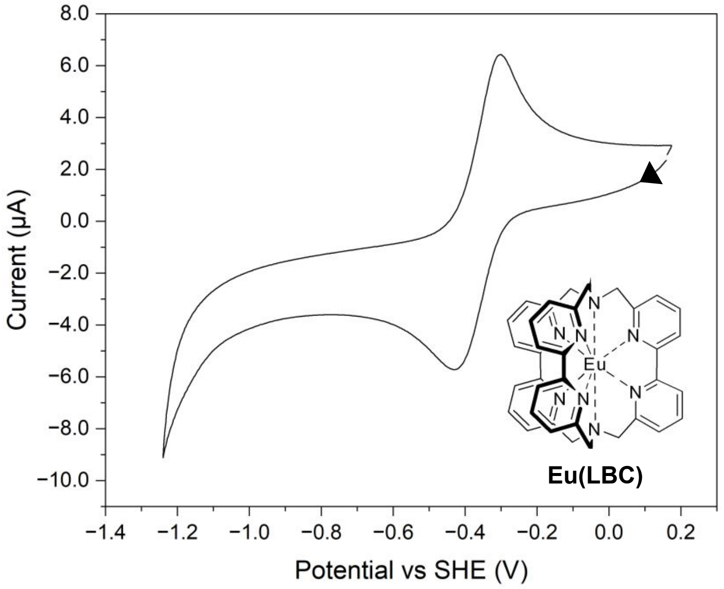

Figure S6: CV of **Eu(LBC)** in 50 mM Tris 50 mM NaCl H_2O at pH 7.4, starting from **Eu^{III}(LBC)**, with scan rate 0.02 V/s scanning cathodically.

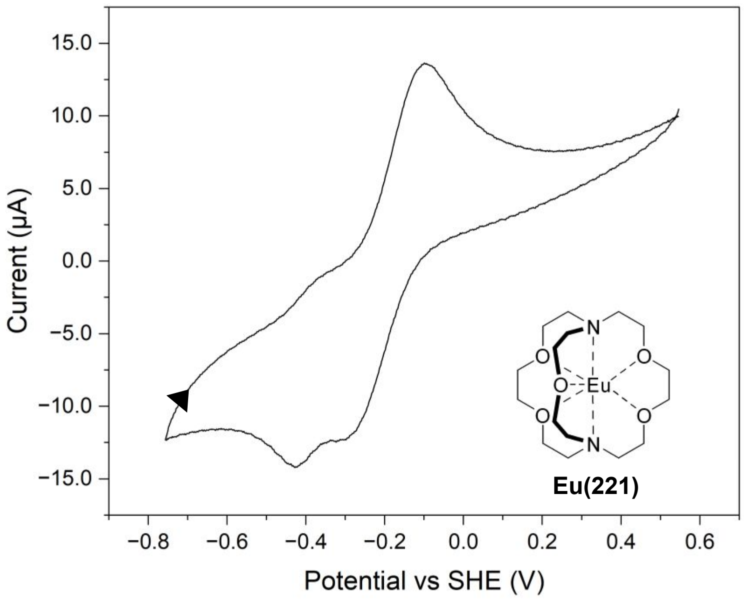

Figure S7: CV of **Eu(221)** in 50 mM Tris 50 mM NaCl H_2O at pH 7.4, starting from **Eu^{II}(221)**, with scan rate 0.02 V/s scanning anodically.

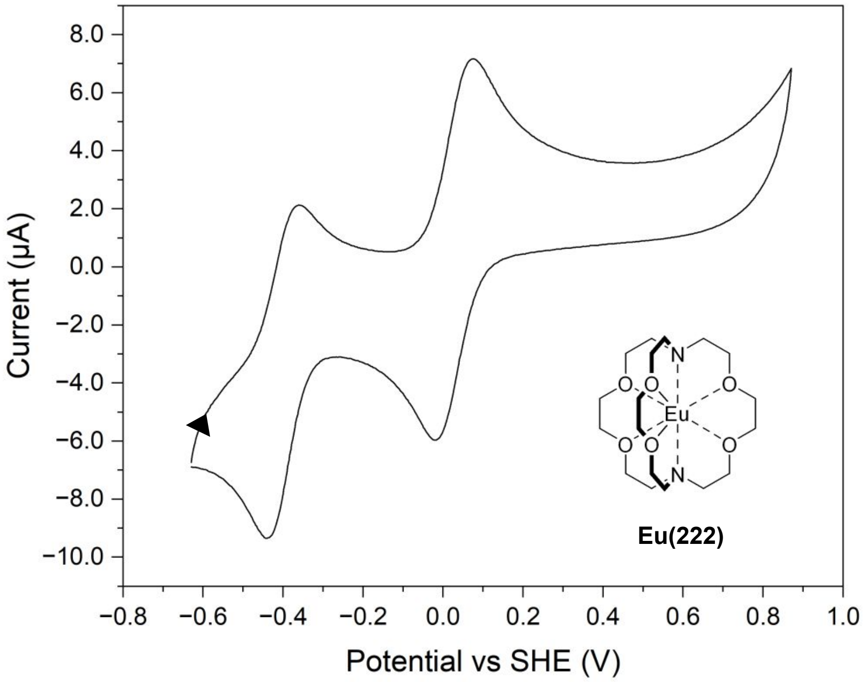

Figure S8: CV of **Eu(222)** in 50 mM Tris 50 mM NaCl H_2O at pH 7.4, starting from **Eu^{II}(222)**, with scan rate 0.02 V/s scanning anodically.

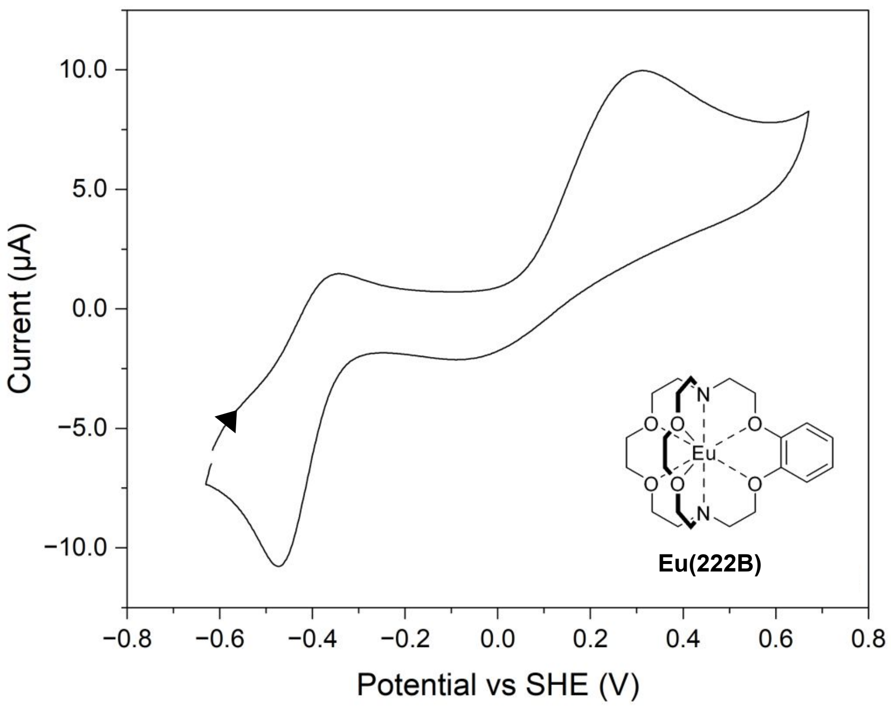

Figure S9: CV of **Eu(222B)** in 50 mM Tris 50 mM NaCl H_2O at pH 7.4, starting from **Eu^{II}(222B)**, with scan rate 0.02 V/s scanning anodically.

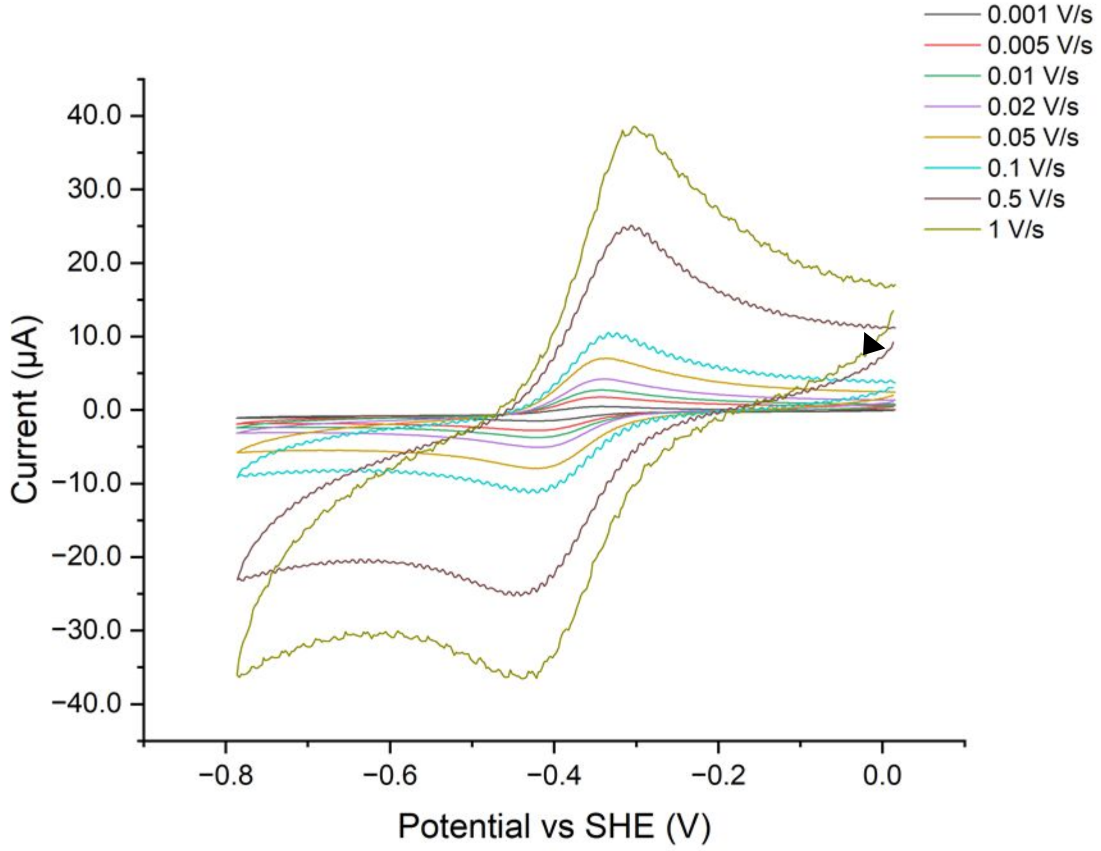

Figure S10: CV of **Eu(LBC)** in 50 mM Tris 50 mM NaCl H_2O at pH 7.4, starting from **Eu^{III}(LBC)**, with scan rate varied scanning cathodically.

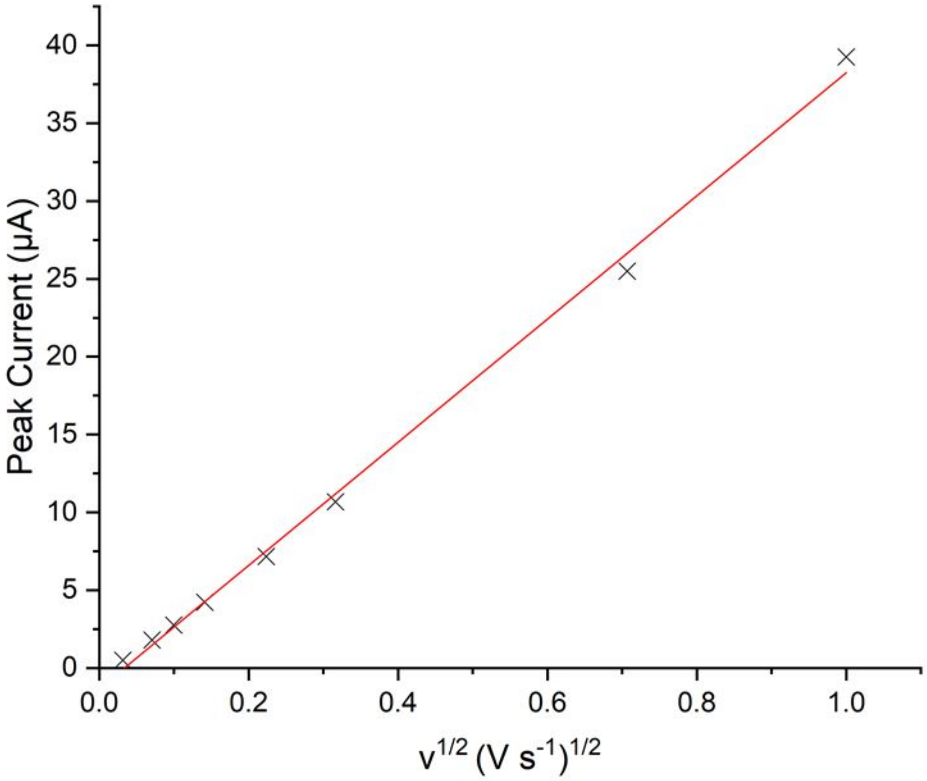

Figure S11: Plot of peak current against square root of scan rate from CVs of **Eu(LBC)** in 50 mM Tris 50 mM NaCl H_2O at pH 7.4, starting from **Eu^{III}(LBC)** and scanning cathodically.

7. T_1 and Relaxivity Measurements

T_1 of water was measured for 0.0, 0.2, 0.4, 0.6, 0.8, and 1.0 mM concentrations on three independently prepared samples of complex (n = 1 - 3) in water at pH 7.4 (50 mM Tris, 50 mM NaCl).

Table S2: T_1 of water values of **Gd^{III}(DOTA)** in H_2O at pH 7.4.

Concentration	T_1 / s (n=1)	T_1 / s (n=2)	T_1 / s (n=3)
0.0	3.544	3.543	3.547
0.2	1.022	1.021	1.022
0.4	0.598	0.597	0.598
0.6	0.412	0.413	0.413
0.8	0.320	0.320	0.320
1.0	0.261	0.261	0.262

Equation of linear fit of mean $1/T_1$ (y) against concentration (x) is y = 0.281 + 3.544x

Table S3: T_1 of water values of **Eu^{II}(LBC)** in H_2O at pH 7.4.

Concentration	T_1 / s (n=1)	T_1 / s (n=2)	T_1 / s (n=3)
0.0	3.650	3.556	3.551
0.2	1.152	1.208	1.215
0.4	0.695	0.683	0.683
0.6	0.473	0.471	0.469
0.8	0.364	0.362	0.359
1.0	0.294	0.294	0.298

Equation of linear fit of mean $1/T_1$ (y) against concentration (x) is y = 0.275 + 3.079x

Table S4: T_1 of water values of **Eu^{III}(LBC)** in H_2O at pH 7.4.

Concentration	T_1 / s (n=1)	T_1 / s (n=2)	T_1 / s (n=3)
0.0	3.613	3.612	3.610
0.2	3.535	3.536	3.534
0.4	3.588	3.588	3.587
0.6	3.548	3.546	3.545
0.8	3.570	3.571	3.572
1.0	3.536	3.520	3.529

Equation of linear fit of mean $1/T_1$ (y) against concentration (x) is y = 0.279 + 0.001x

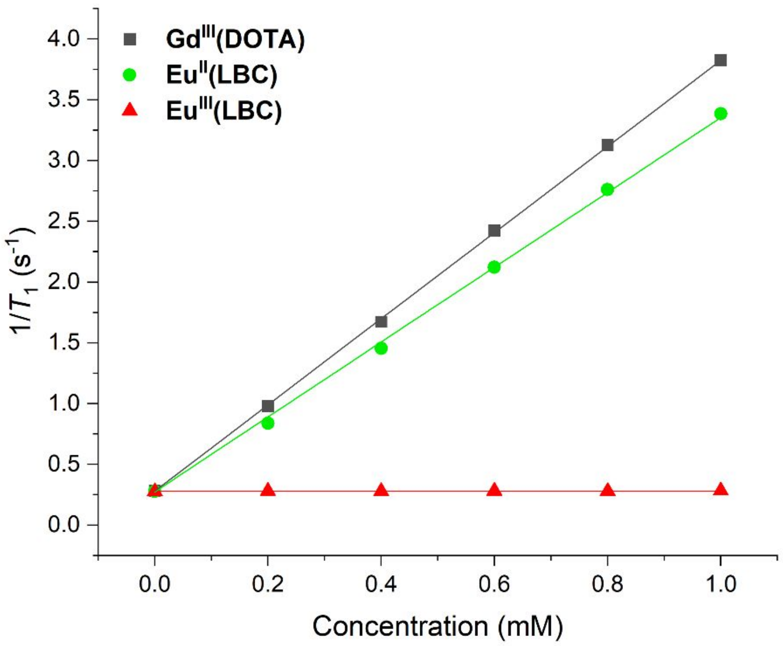

Figure S12: 1/T_1 versus concentration plot for **Eu^{III}(LBC)**, **Eu^{II}(LBC)** and **Gd^{III}(DOTA)** measured in degassed H_2O at pH 7.4 (50 mM Tris, 50 mM NaCl) using an 11.75 T (499.9 MHz) NMR spectrometer and the corresponding linear fits.

Table S5: the tabulated T_1 relaxivity values (499.9 MHz) for each complex in degassed H_2O at pH 7.4 (50 mM Tris, 50 mM NaCl).

Complex	T_1 relaxivity, r_1 / mM^{-1} s^{-1}
**Gd^{III}(DOTA)**	3.54 ± 0.02
**Eu^{II}(LBC)**	3.08 ± 0.03
**Eu^{III}(LBC)**	0.001 ± 0.004

8. Enzyme/Cofactor Assays

All enzymatic assays were carried out in degassed water at pH 7.4 (50 mM Tris, 50 mM NaCl) with 1 mM of **Eu^{III}(LBC)**, 4 μM of enzyme, and 10 mM of cofactor at 37 ^{o}C with gentle stirring using 2 mm magnetic stirrer balls for proteins in a glove box under a N_2 atmosphere (O_2 < 2 ppm). For the assays involving Hyd-1 and Hyd-2, the enzyme was activated prior to use in the assay by placing the enzyme in a pressure vessel under 1 bar of H_2 gas for 4 h; H_2 gas was gently bubbled through the reaction mixture throughout the assay. Cyt c $_{red..}$ was obtained by reducing oxidised cytochrome c (cyt c $_{ox.}$) with $Na_2S_2O_4$ (1 mM cyt c $_{ox.}$:10 mM $Na_2S_2O_4$); the reduction was followed by UV-Vis absorbance spectroscopy by monitoring growth of the peaks at 520 and 550 nm;[17] once reduction was complete, the $Na_2S_2O_4$ was removed using a Amicon® Ultra Centrifugal Filter, 3 kDa MWCO (13.4 krpm for 20 min). For the assays involving cyt c, 1 mM cyt c $_{red.}$:1 mM **Eu^{III}(LBC)** and 1 mM cyt c $_{ox.}$:1 mM **Eu^{II}(LBC)** were used. To aerate a sample of **Eu^{II}(LBC)**, a 1 mM solution was removed from the glove box where it was synthesised (O_2 < 2 ppm) and gently shaken open to air for 5 min. $FMNH_2$ and $FADH_2$ were obtained by adapting a reported procedure,[18,19] which involved reducing FMN or FAD with the Hyd-1 enzyme and H_2 gas: Hyd-1 (4 μM) and FMN or FAD (1 mM) were combined in degassed water at pH 7.4 (50 mM Tris, 50 mM NaCl) and H_2 gas was gently bubbled through the reaction mixture within a glove box of N_2 atmosphere (O_2 < 2 ppm) for 4 h; reduction of FMN and FAD to $FMNH_2$ and $FADH_2$ was confirmed by UV-Visible absorbance spectroscopy through loss of absorbance at 445 nm and 450 nm, respectively;[18,19] the Hyd-1 enzyme was then removed using a Amicon® Ultra Centrifugal Filter, 3 kDa MWCO (13.4 krpm for 30 min); for the assays, 1 mM $FMNH_2$:1 mM **Eu^{III}(LBC)** and 1 mM $FADH_2$:1 mM **Eu^{III}(LBC)** were used. For all assays, after 12 h, T_1 of water was measured three times on the reactions (unless stated otherwise) and controls in melting point tubes within a Young's Tap NMR tube, all sealed in a glove box under N_2 atmosphere (O_2 < 2 ppm) prior to removal for measuring.

Table S6: T_1 of water values in HoxFU/NADH assay.

Reaction/Control	T_1 / s	T_1 / s	T_1 / s	Mean T_1 / s
**Eu^{III}(LBC)** + HoxFU + NADH	0.509	0.509	0.510	0.509(1)
HoxFU + NADH	3.497	3.495	3.500	3.497(1)
**Eu^{III}(LBC)** + NADH	3.349	3.347	3.353	3.350(1)

Table S7: T_1 of water values in Hyd-1/H_2 assay.

Reaction/Control	T_1 / s	T_1 / s	T_1 / s	Mean T_1 / s

**Eu^{III}(LBC)** + Hyd-1 + H_2	0.626	0.856	0.826	0.77(6)
Hyd-1 + H_2	3.504	3.449	3.450	3.45(2)
**Eu^{III}(LBC)** + H_2	3.418	3.419	3.421	3.419(1)

Table S8: T_1 of water values in Hyd-2/H_2 assay.

Reaction/Control	T_1 / s	T_1 / s	T_1 / s	Mean T_1 / s
**Eu^{III}(LBC)** + Hyd-2 + H_2	0.3398	0.3397	0.3388	0.3394(3)
Hyd-2 + H_2	3.234	3.239	3.237	3.236(2)

Table S9: T_1 of water values in POR/NADPH assay.

Reaction/Control	T_1 / s	T_1 / s	T_1 / s	Mean T_1 / s
**Eu^{III}(LBC)** + POR + NADPH	3.171	3.171	3.165	3.169(2)
POR + NADPH	3.369	3.378	3.372	3.373(3)
**Eu^{III}(LBC)** + NADPH	3.358	3.366	3.359	3.361(3)

Table S10: T_1 of water values in FNR/NADPH assay.

Reaction/Control	T_1 / s	T_1 / s	T_1 / s	Mean T_1 / s
**Eu^{III}(LBC)** + FNR + NADPH	2.895	2.887	2.884	2.889(3)
FNR + NADPH	3.251	3.250	3.254	3.252(1)

Table S11: T_1 of water values in cyt c $_{(red.)}$ assay.

Reaction/Control	T_1 / s	T_1 / s	T_1 / s	Mean T_1 / s
**Eu^{III}(LBC)** + cyt c $_{red.}$	3.309	3.309	3.306	3.308(1)
cyt c $_{red.}$	3.374	3.374	3.375	3.374(1)

Table S12: T_1 of water values in $FMNH_2$ assay.

Reaction/Control	T_1 / s	T_1 / s	T_1 / s	Mean T_1 / s
**Eu^{III}(LBC)** + $FMNH_2$	3.270	3.269	3.275	3.271(2)

$FMNH_2$	3.349	3.349	3.348	3.349(1)

Table S13: T_1 of water values in $FADH_2$ assay.

Reaction/Control	T_1 / s	T_1 / s	T_1 / s	Mean T_1 / s
**Eu^{III}(LBC)** + $FADH_2$	3.129	3.129	3.131	3.130(1)
$FADH_2$	3.267	3.266	3.267	3.267(1)

Table S14: T_1 of water values in HoxFU/NAD^+ assay.

Reaction/Control	T_1 / s	T_1 / s	T_1 / s	Mean T_1 / s
**Eu^{II}(LBC)** + HoxFU + NAD^+	2.739	2.749	2.776	2.755(9)
HoxFU + NAD^+	3.096	3.096	3.099	3.097(1)
**Eu^{II}(LBC)** + NAD^+	0.471	0.471	0.471	0.471(1)

Table S15: T_1 of water values in aeration assay.

Reaction/Control	T_1 / s	T_1 / s	T_1 / s	Mean T_1 / s
**Eu^{II}(LBC)** + air*	2.918	2.925	2.934	2.926(4)

*For this assay, a 1 mM solution of **Eu^{II}(LBC)** was exposed to air for 5 minutes, then the T_1 value of the solution was measured immediately thereafter.

Table S16: T_1 of water values in Hyd-2/H^+ assay.

Reaction/Control	T_1 / s	T_1 / s	T_1 / s	Mean T_1 / s
**Eu^{II}(LBC)** + Hyd-2 + N_2 *	1.548	1.555	1.571	1.558(6)
Hyd-2 + N_2 *	3.145	3.149	3.151	3.148(2)

*These reactions were a direct follow on from the Hyd-2/H_2 assay, starting with 86.8% **Eu^{2+}(LBC)** after conversion of **Eu^{III}(LBC)** to **Eu^{II}(LBC)** by Hyd-2/H_2.

Table S17: T_1 of water values in POR/$NADP^+$ assay.

Reaction/Control	T_1 / s	T_1 / s	T_1 / s	Mean T_1 / s
**Eu^{II}(LBC)** + POR + $NADP^+$	3.296	3.296	3.296	3.296(1)
POR + $NADP^+$	3.184	3.181	3.181	3.182(1)

**Eu^{II}(LBC)** + NADP$^+$	0.668	0.669	0.669	0.669(1)

Table S18: T_1 of water values in FNR/NADP$^+$ assay.

Reaction/Control	T_1 / s	T_1 / s	T_1 / s	Mean T_1 / s
**Eu^{II}(LBC)** + FNR + NADP$^+$	3.316	3.311	3.320	3.315(4)
FNR + NADP$^+$	3.475	3.474	3.478	3.476(1)

Table S19: T_1 of water values in cyt c $_{(ox.)}$ assay.

Reaction/Control	T_1 / s	T_1 / s	T_1 / s	Mean T_1 / s
**Eu^{II}(LBC)** + cyt c $_{ox.}$	3.215	3.218	3.221	3.218(2)
cyt c $_{ox.}$	3.228	3.228	3.229	3.228(1)

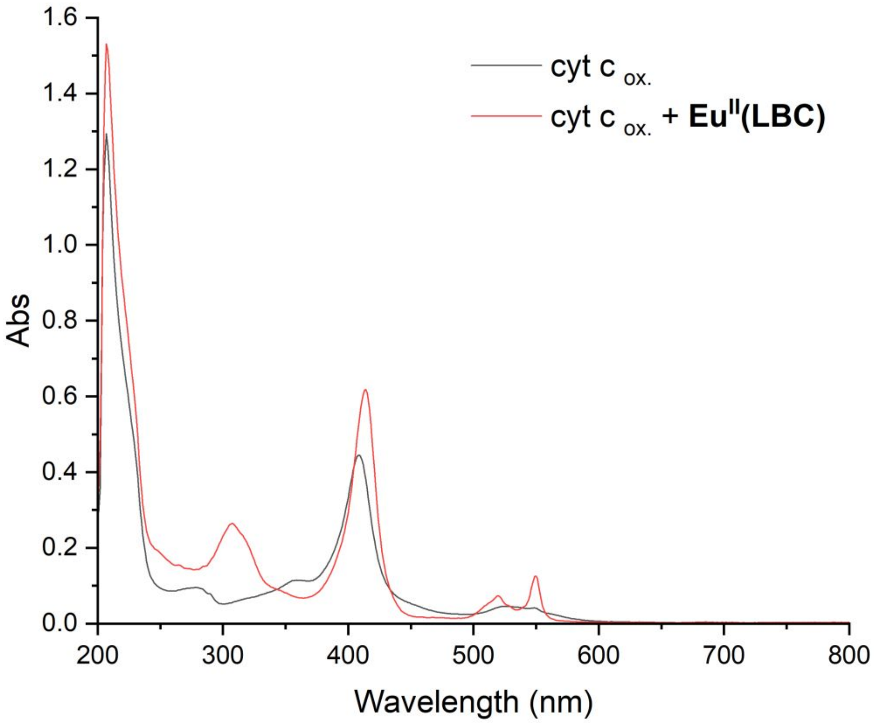

Figure S13: The UV-Vis absorbance spectra of cyt c $_{red.}$ and cyt c $_{ox.}$ + **Eu^{II}(LBC)** in degassed H_2O at pH 7.4 (50 mM Tris, 50 mM NaCl) at concentration of 5 μM.

Table S20: T_1 of water values in FMN assay.

Reaction/Control	T_1 / s	T_1 / s	T_1 / s	Mean T_1 / s
**Eu^{II}(LBC)** + FMN	3.399	3.397	3.398	3.398(1)
FMN	3.176	3.179	3.180	3.178(1)

Table S21: T_1 of water values in FAD assay.

Reaction/Control	T_1 / s	T_1 / s	T_1 / s	Mean T_1 / s
**Eu^{II}(LBC)** + FAD	3.441	3.444	3.442	3.442(1)
FAD	3.550	3.550	3.551	3.550(1)

Table S22: T_1 of water values in GSH assay.

Reaction/Control	T_1 / s	T_1 / s	T_1 / s	Mean T_1 / s
**Eu^{III}(LBC)** + GSH	3.394	3.394	3.391	3.393(1)
GSH	3.534	3.536	3.536	3.535(1)

9. Nernstian Calculation

$$2\boldsymbol{Eu^{III}(LBC)} + NAD(P)H \rightleftharpoons 2\boldsymbol{Eu^{II}(LBC)} + NAD(P)^{+} + H^{+}$$

$$At\ pH\ 7.4\colon E_{cathode} = -0.360\ V,\ E_{anode} = -0.332\ V$$

$$E_{cell} = E_{cathode} - E_{anode} = -0.360\ V - (-0.332\ V) = -0.028\ V = \frac{RT}{nF} lnK$$

$$\Rightarrow lnK = \frac{-0.028\ V(2)\ (96{,}485\ Cmol^{-1})}{8.314\ JK^{-1}mol^{-1}(298\ K)} = -2.180\ldots$$

$$\Rightarrow K(pH\ 7.4) = 0.112\ldots = \frac{[\boldsymbol{Eu^{II}(LBC)}]^{2}[NAD(P)^{+}]}{[\boldsymbol{Eu^{III}(LBC)}]^{2}[NAD(P)H]}$$

	Initial Concentration / M	Change in Concentration / M	Equilibrium Concentration / M
$\boldsymbol{Eu^{III}(LBC)}$	1×10^{-3}	$-2x$	$1 \times 10^{-3} - 2x$
$NAD(P)H$	10×10^{-3}	$-x$	$10 \times 10^{-3} - x$
$\boldsymbol{Eu^{II}(LBC)}$	0	$+2x$	$2x$
$NAD(P)^{+}$	0	$+x$	x

$$\Rightarrow K(pH\ 7.4) = \frac{(2x)^{2}(x)}{(1 \times 10^{-3} - 2x)^{2}(10 \times 10^{-3} - x)} = 0.112\ldots$$

$$\Rightarrow x = 0.000323\ldots$$

	Equilibrium Concentration / 10^{-3} M
$\boldsymbol{Eu^{III}(LBC)}$	0.352…
$NAD(P)H$	9.676…
$\boldsymbol{Eu^{II}(LBC)}$	0.647…
$NAD(P)^{+}$	0.323…

$$\Rightarrow\ \approx 65\%\ conversion\ of\ \boldsymbol{Eu^{III}(LBC)}\ to\ \boldsymbol{Eu^{II}(LBC)}$$

10. NMR Spectra

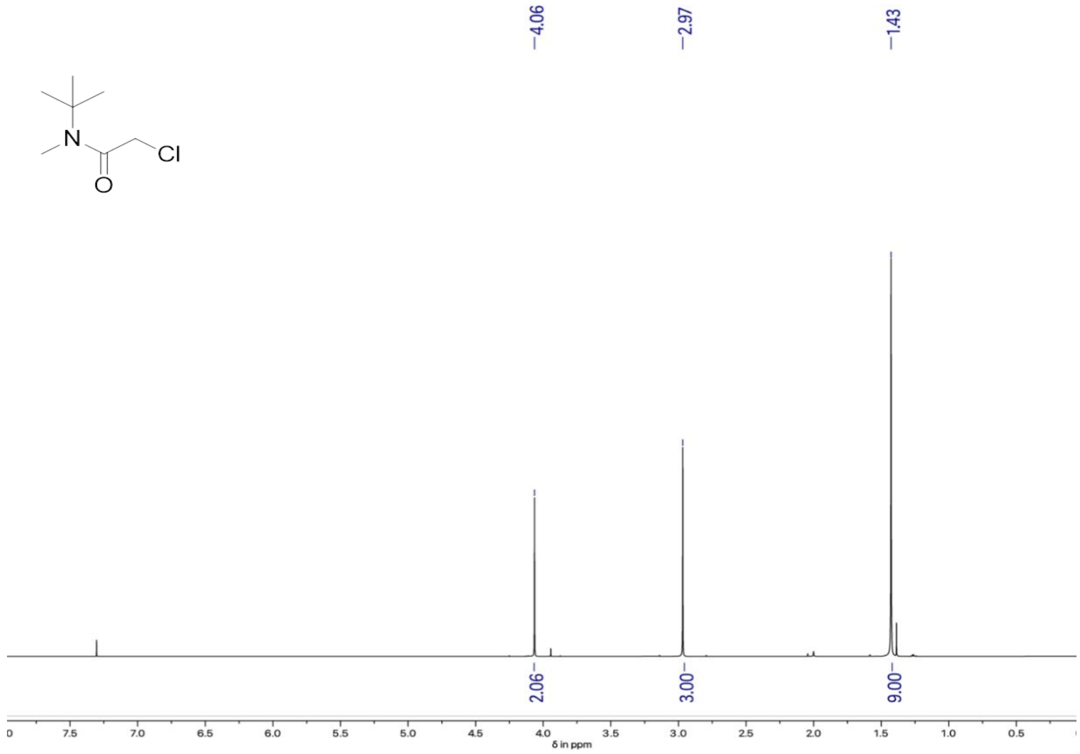

Figure S14: 400.2 MHz ^{1}H NMR spectrum of **TAC.Arm** in $CDCl_3$.

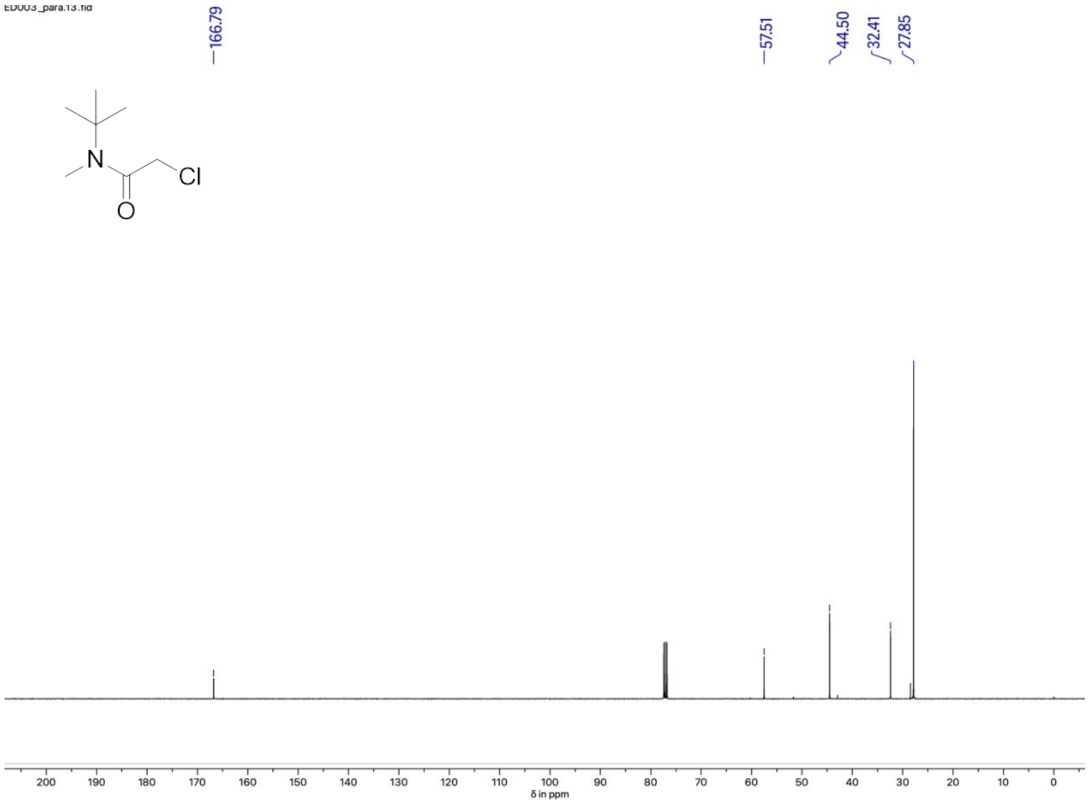

Figure S15: 100.6 MHz ^{13}C NMR spectrum of **TAC.Arm** in $CDCl_3$.

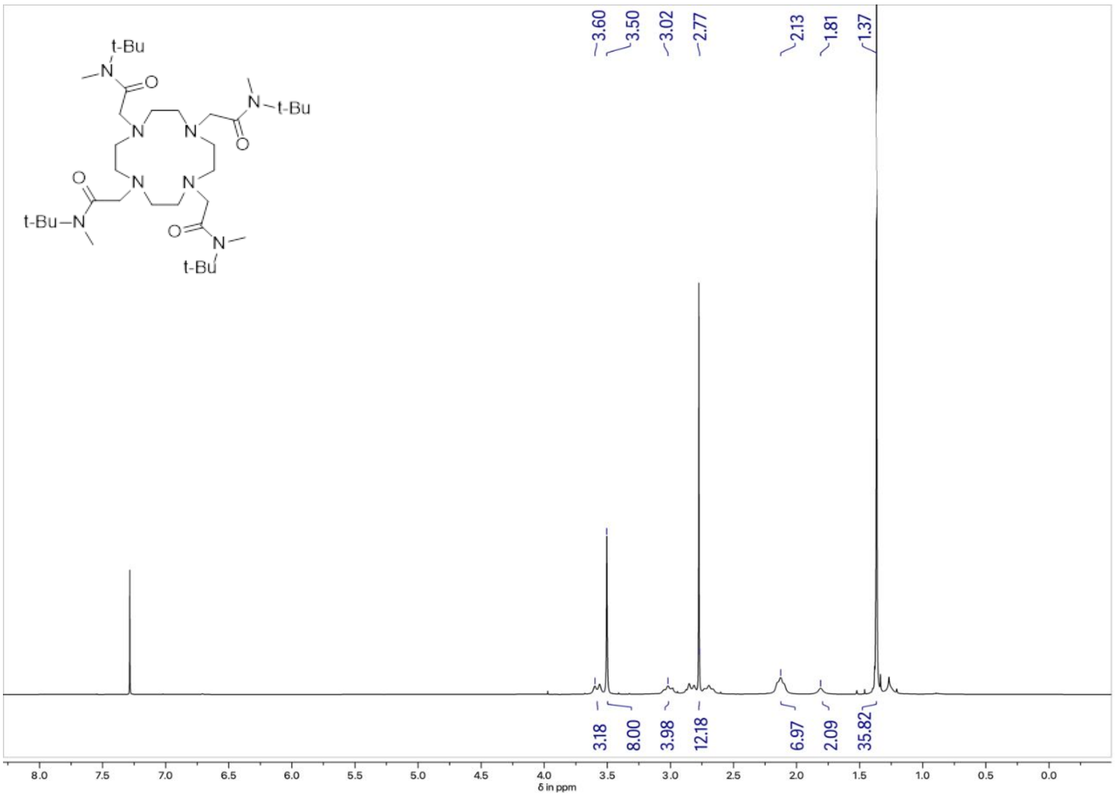

Figure S16: 400.2 MHz ^{1}H NMR spectrum of **TAC** in $CDCl_3$.

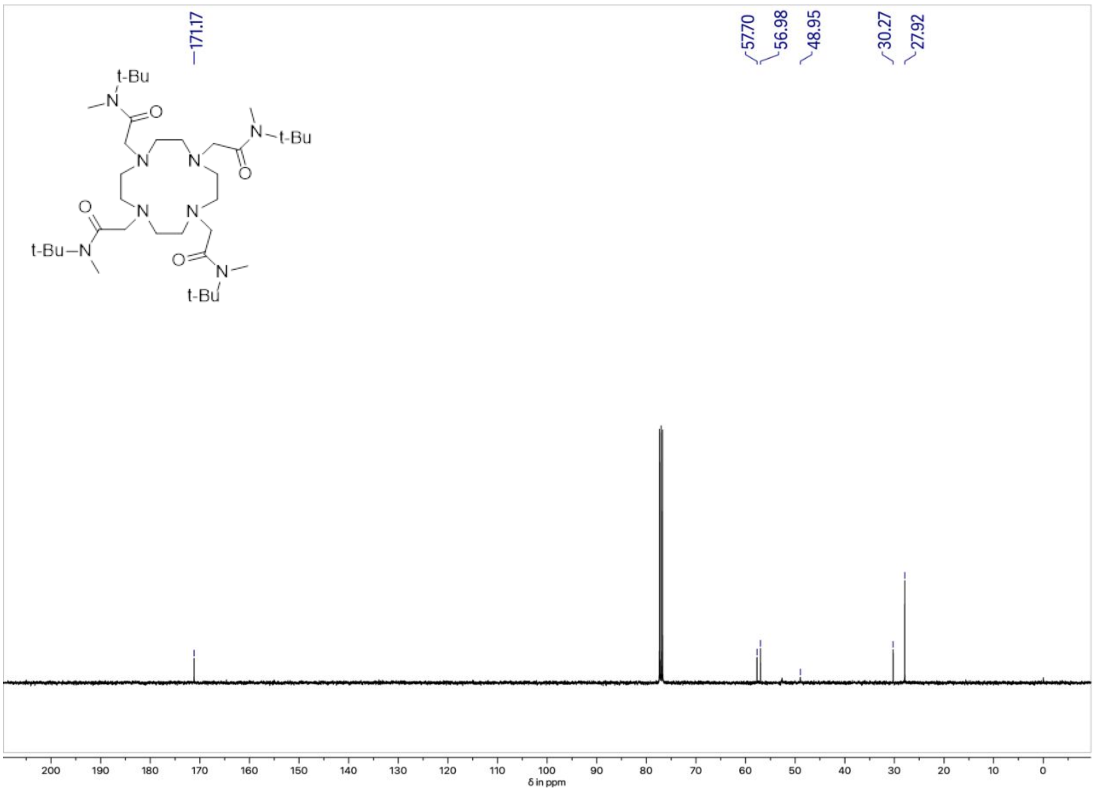

Figure S17: 100.6 MHz ^{13}C NMR spectrum of **TAC** in $CDCl_3$.

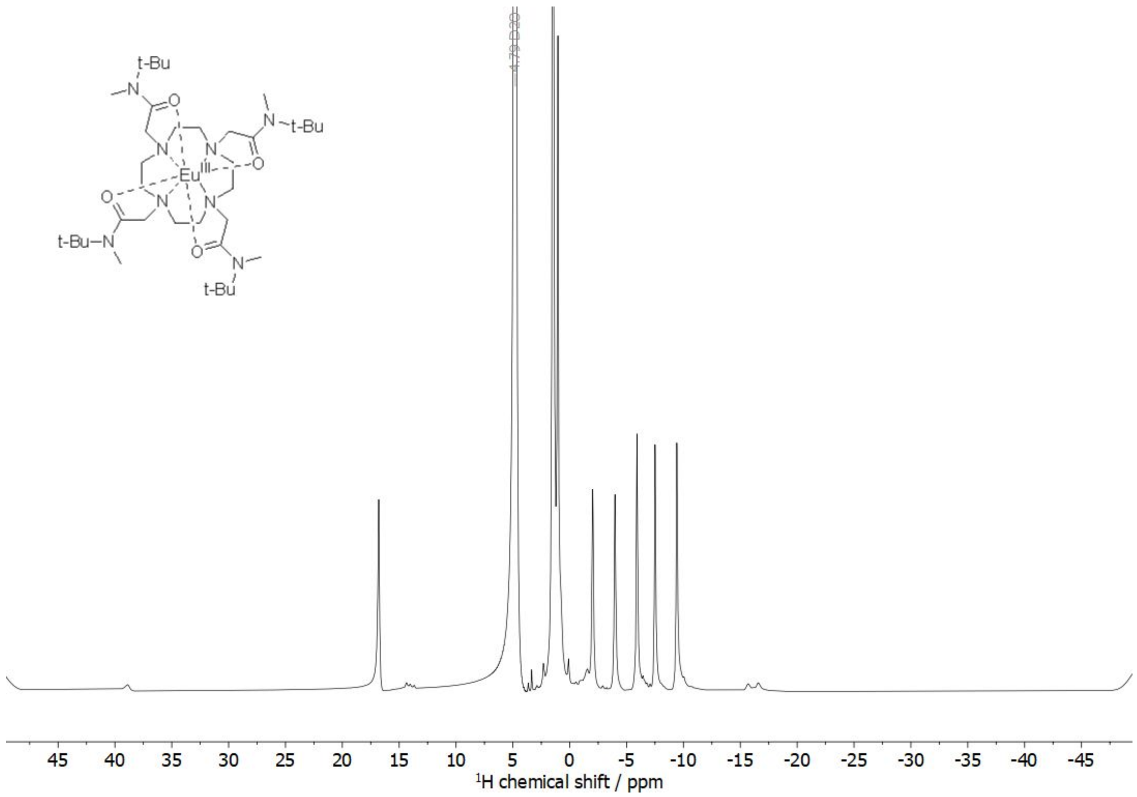

Figure S18: 400.2 MHz ^{1}H NMR spectrum of **Eu^{III}(TAC)** in D_2O.

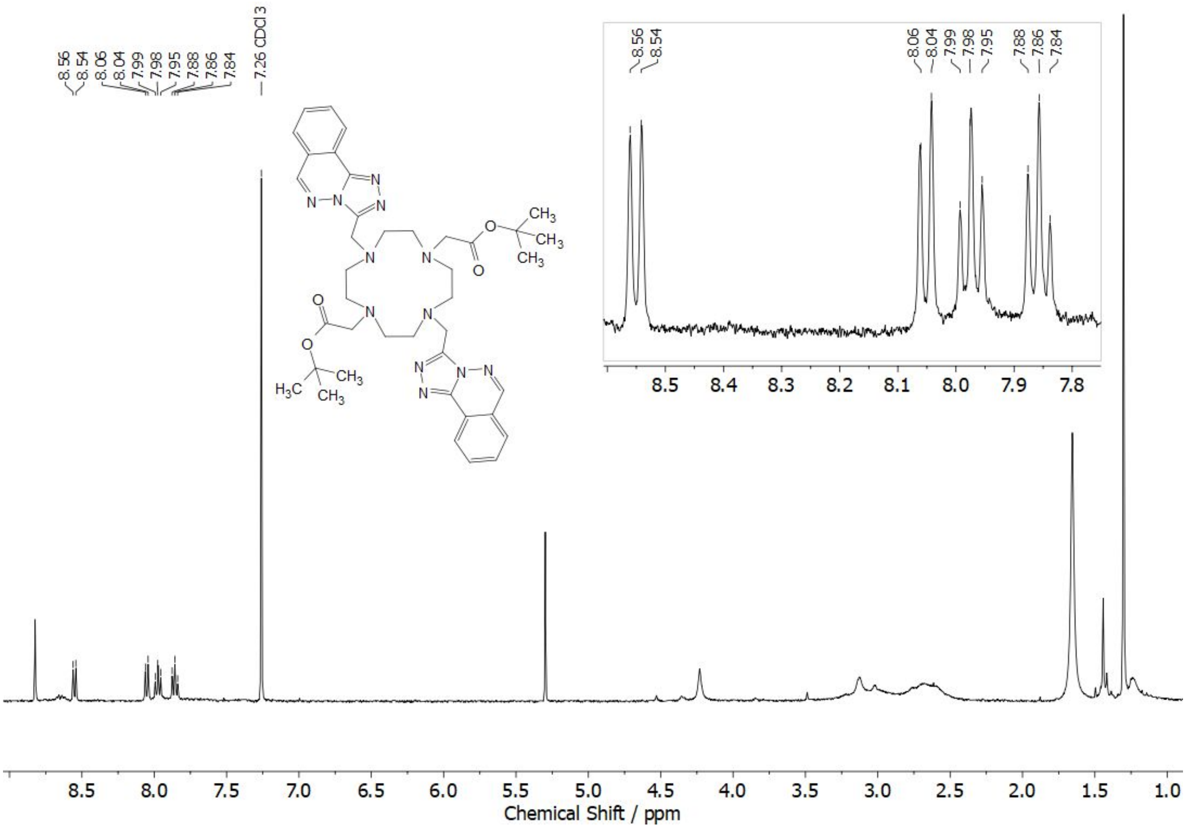

Figure S19: 400.2 MHz ^{1}H NMR spectrum of **DTDCC.P4** in $CDCl_3$.

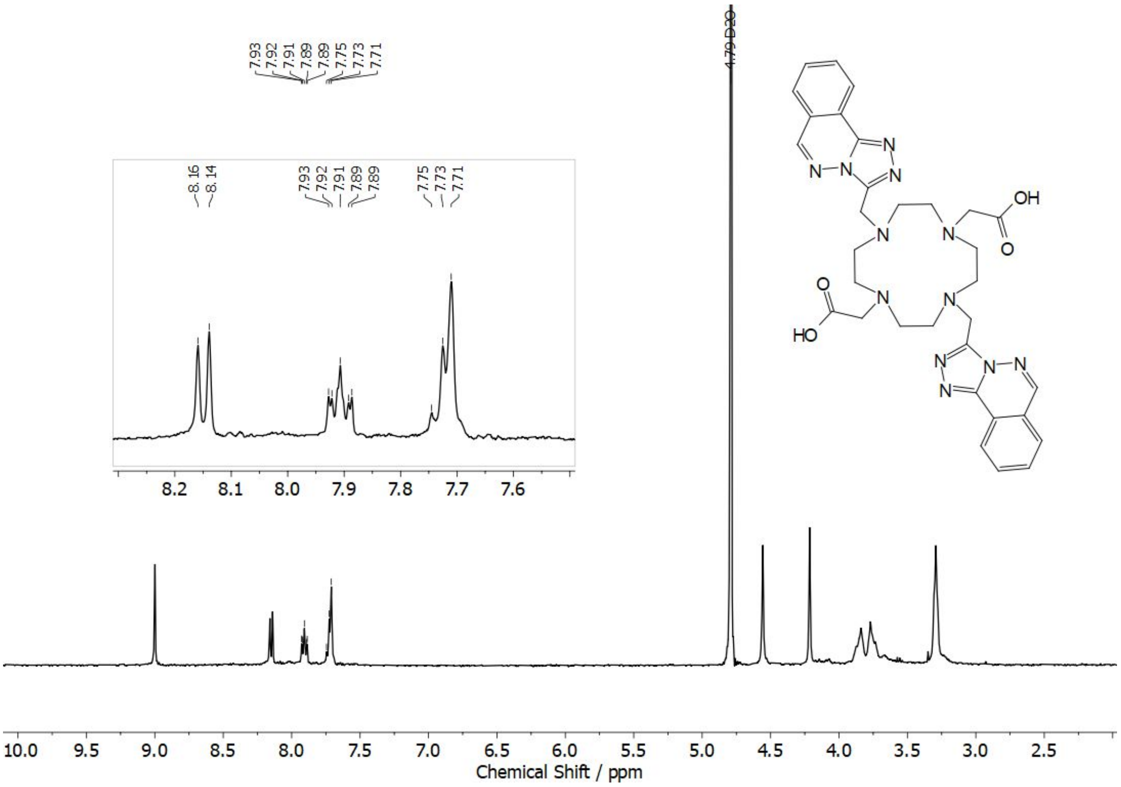

Figure S20: 400.2 MHz ^{1}H NMR spectrum of **DTDCC** in D_2O.

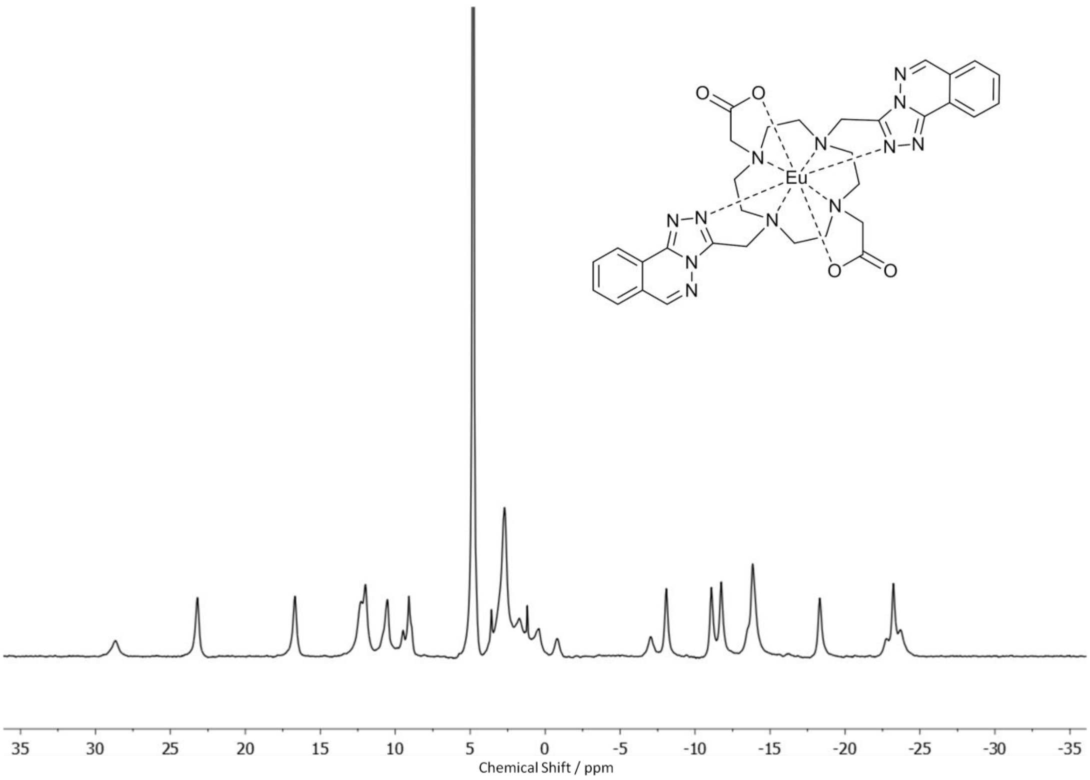

Figure S21: 400.2 MHz ^{1}H NMR spectrum of **Eu^{III}(DTDCC)** in D_2O.

References

(1) Lauterbach, L.; Idris, Z.; Vincent, K. A.; Lenz, O. Catalytic Properties of the Isolated Diaphorase Fragment of the NAD +-Reducing [NiFe]-Hydrogenase from Ralstonia Eutropha. *PLoS One* **2011**, *6* (10), p.e25939. DOI: 10.1371/journal.pone.0025939

(2) Evans, R. M.; Brooke, E. J.; Wehlin, S. A. M.; Nomerotskaia, E.; Sargent, F.; Carr, S. B.; Phillips, S. E. V.; Armstrong, F. A. Mechanism of Hydrogen Activation by [NiFe] Hydrogenases. *Nat. Chem. Biol.* **2016**, *12* (1), 46–50. DOI: 10.1038/nchembio.1976

(3) Beaton, S. E.; Evans, R. M.; Finney, A. J.; Lamont, C. M.; Armstrong, F. A.; Sargent, F.; Carr, S. B. The Structure of Hydrogenase-2 from Escherichia Coli: Implications for H2-Driven Proton Pumping. *Biochem. J.* **2018**, *475* (7), 1353–1370. DOI: 10.1042/BCJ20180053

(4) Siritanaratkul, B.; Megarity, C. F.; Roberts, T. G.; Samuels, T. O. M.; Winkler, M.; Warner, J. H.; Happe, T.; Armstrong, F. A. Transfer of Photosynthetic NADP+/NADPH Recycling Activity to a Porous Metal Oxide for Highly Specific, Electrochemically-Driven Organic Synthesis. *Chem. Sci.* **2017**, *8* (6), 4579–4586. DOI: 10.1039/c7sc00850c

(5) Rodriguez-Maciá, P.; Dutta, A.; Lubitz, W.; Shaw, W. J.; Rüdiger, O. Direct Comparison of the Performance of a Bio-inspired Synthetic Nickel Catalyst and a [NiFe]-Hydrogenase, Both Covalently Attached to Electrodes. *Angew. Chem. Int. Ed.* **2015**, *127* (42), 12478–12482. DOI: 10.1002/anie.201502364

(6) Marques, M. P. M.; Geraldes, C. F. G. C.; Sherry, A. D.; Merbach, A. E.; Powell, H.; Pubanz, D.; Aime, S.; Botta, M. NMR Conformational Study of The Lanthanide(III) Complexes of DOTA in Aqueous Solution. *J. Alloys. Compd.* **1995**, *225* (1-2), 303-307. DOI: 10.1016/0925-8388(94)07083-0

(7) Burton-Pye, B. P.; Heath, S. L.; Faulkner, S. Synthesis and Luminescence Properties of Lanthanide Complexes Incorporating a Hydralazine-Derived Chromophore. *Dalton Trans.* **2005**, No. 1, 146–149. DOI: 10.1039/b413005g

(8) Harnden, A.C.; Suturina, E.A.; Batsanov, A.S.; Senanayake, P.K.; Fox, M.A.; Mason, K.; Vonci, M.; McInnes, E.J.; Chilton, N.F.; Parker, D., Unravelling the complexities of pseudocontact shift analysis in lanthanide coordination complexes of differing symmetry. *Angew. Chem. Int. Ed.* **2019**, *131* (30), 10396-10400. DOI: 10.1002/ange.201906031

(9) Natrajan, L. S.; Khoabane, N. M.; Dadds, B. L.; Muryn, C. A.; Pritchard, R. G.; Heath, S. L.; Kenwright, A. M.; Kuprov, I.; Faulkner, S. Probing the Structure, Conformation, and Stereochemical Exchange in a Family of Lanthanide Complexes Derived from Tetrapyridyl-Appended Cyclen. *Inorg. Chem.* **2010**, *49* (17), 7700–7709. DOI: 10.1021/ic100447m

(10) Routledge, J. D. Exploring Interactions between Anions and Kinetically Stable Lanthanide Complexes in Aqueous Solution, University of Oxford, 2016. https://ora.ox.ac.uk/objects/uuid:69e73701-0689-475a-ac33-ee260fa8baea

(11) Nardi, G.; Lineros-Rosa, M.; Palumbo, F.; Miranda, M. A.; Lhiaubet-Vallet, V. Spectroscopic Characterization of Dipicolinic Acid and Its Photoproducts as Thymine Photosensitizers. *Spectrochim. Acta A Mol. Biomol. Spectrosc.* **2021**, *245*. DOI: 10.1016/j.saa.2020.118898

(12) Li, W.; Xie, J. H.; Yuan, M. L.; Zhou, Q. L. Ruthenium Complexes of Tetradentate Bipyridine Ligands: Highly Efficient Catalysts for the Hydrogenation of Carboxylic Esters and Lactones. *Green Chem.* **2014**, *16* (9), 4081–4085. DOI: 10.1039/c4gc00835a

(13) Rodriguz-Ubis, J. -C; Alpha, B.; Plancherel, D.; Lehn, J. -M. Photoactive Cryptands. Synthesis of the Sodium Cryptates of Macrobicyclic Ligands Containing Bipyridine and Phenoanthroline Groups. *Helv. Chim. Acta* **1984**, *67* (8), 2264–2269. DOI: 10.1002/hlca.19840670833

(14) Alpha, B.; Lehn, J. -M; Mathis, G. Energy Transfer Luminescence of Europium(III) and Terbium(III) Cryptates of Macrobicyclic Polypyridine Ligands. *Angew. Chem. Int. Ed.* **1987**, *26* (3), 266–267. DOI: 10.1002/anie.198702661.

(15) Gamage, N. D. H.; Mei, Y.; Garcia, J.; Allen, M. J. Oxidatively Stable, Aqueous Europium(II) Complexes through Steric and Electronic Manipulation of Cryptand Coordination Chemistry. *Angew. Chem. Int. Ed.* **2010**, *49* (47), 8923–8925. DOI: 10.1002/anie.201002789

(16) Burnett, M. E.; Adebesin, B.; Funk, A. M.; Kovacs, Z.; Sherry, A. D.; Ekanger, L. A.; Allen, M. J.; Green, K. N.; Ratnakar, S. J. Electrochemical Investigation of the Eu3+/2+ Redox Couple in Complexes with Variable Numbers of Glycinamide and Acetate Pendant Arms. *Eur. J. Inorg. Chem.* **2017**, *2017* (43), 5001–5005. DOI: 10.1002/ejic.201701070

(17) Rodríguez-Roldán, V.; García-Heredia, J. M.; Navarro, J. A.; Hervás, M.; la Cerda, B. De; Molina-Heredia, F. P.; De la Rosa, M. A. A Comparative Kinetic Analysis of the Reactivity of Plant, Horse, and Human Respiratory Cytochrome c towards Cytochrome c Oxidase. *Biochem. Biophys. Res. Commun.* **2006**, *346* (3), 1108–1113. DOI: 10.1016/j.bbrc.2006.06.022

(18) Joseph Srinivasan, S.; Cleary, S.E.; Ramirez, M.A.; Reeve, H.A.; Paul, C.E.; Vincent, K.A. E. coli Nickel-Iron Hydrogenase 1 Catalyses Non-native Reduction of Flavins: Demonstration for Alkene Hydrogenation by Old Yellow Enzyme Ene-reductases. *Angew. Chem. Int. Ed.*, *133* (25), 13943-13947. DOI: 10.1002/ange.202101186

(19) Ramirez, M. A.; Joseph Srinivasan, S.; Cleary, S. E.; Todd, P. M. T.; Reeve, H. A.; Vincent, K. A. H_2-Driven Reduction of Flavin by Hydrogenase Enables Cleaner Operation of Nitroreductases for Nitro-Group to Amine Reductions. *Front. Catal.* **2022**, *2*, p.906694. DOI: 10.3389/fctls.2022.906694
